# Oncotype Dx Score, HER2 Low Expression, and Clinical Outcomes in Early-Stage Breast Cancer: A National Cancer Database Analysis

**DOI:** 10.3390/cancers15174264

**Published:** 2023-08-25

**Authors:** Arya Mariam Roy, Changchuan Jiang, Stuthi Perimbeti, Lei Deng, Charles L. Shapiro, Shipra Gandhi

**Affiliations:** 1 Department of Medicine, Roswell Park Comprehensive Cancer Center, Buffalo, NY 14203, USA; arya.roy@roswellpark.org (A.M.R.); changchuan.jiang@roswellpark.org (C.J.); stuthi.perimbeti@roswellpark.org (S.P.); lei.deng@roswellpark.org (L.D.); 2 Division of Hematology and Medical Oncology, Department of Medicine, Icahn School of Medicine at Mount Sinai, New York, NY 10029, USA; charles.shapiro@mssm.com

**Keywords:** HER2-low breast cancer, HER2-zero breast cancer, Oncotype Dx, hormone-positive breast cancer, hormone-negative breast cancer, anti-HER2 agents

## Abstract

**Simple Summary:**

The relation between HER2-low status in breast cancer and the hormone receptor-positive breast cancer recurrence score—oncotype recurrence score—is not very well studied. We conducted a study to look at the utility of the oncotype recurrence score in HER2-low and HER2-zero breast cancer by using the data from the National Cancer Database. We found that the recurrence score of HER2-low breast cancer was slightly higher compared to HER2-zero breast cancer. Women with HER2-low breast cancer had better survival, especially for those with hormone receptor-negative breast cancer. Among those who received chemotherapy for breast cancer with a high recurrence score, those with HER2-low breast cancer had higher survival compared to HER2-zero breast cancer.

**Abstract:**

Background: The interaction between HER2-low expression, oncotype recurrence score (RS), and their influence on the prognosis of HR+/HER2- breast cancer (BC) is not very well studied. Methods: We conducted a retrospective cohort study of patients diagnosed with resectable HER2-low and HER2-zero BC from the National Cancer Database. The primary outcome was overall survival (OS), and the association of RS with the clinical outcomes in HR+/HER2- BC was analyzed as an exploratory endpoint. Results: The distribution of RS was comparable between HER2-low and HER2-zero groups; however, the RSs of HER2-low tumors were more likely to be 16–25. Women with HER2-low tumors had longer 5-year OS than women with HER2-zero tumors in the HR-negative (84.3% vs. 83.9%; *p* < 0.001, HR: 0.87 (0.84–0.90), *p* < 0.001) but not in the HR-positive group (94.0% vs. 94.0%; *p* = 0.38, HR: 0.97 (0.95–0.99), *p* = 0.01). The survival advantage was observed in patients who received adjuvant/neoadjuvant chemotherapy (p-interaction (chemo vs. no chemo) < 0.001). Among those who received adjuvant chemotherapy in the group with higher RSs (26–100), those with HER2-low BC had higher 5-year OS than HER2-zero BC. Conclusions: Resectable HER2-low BC had a better prognosis than HER2-zero BC. Among those who received adjuvant chemotherapy in the higher oncotype RS group, those with HER2-low tumors had better survival.

## 1. Introduction

HER2 is a critical oncogene and well-established therapeutic target in breast cancer and other cancers [1]. Over the past twenty years, anti-HER2 therapies such as trastuzumab, pertuzumab, ado-trastuzumab emtansine (T-DM1) revolutionized the treatment landscape of resectable HER2-overexpressing breast cancers [2,3,4]. Conventional anti-HER2 targeted treatment did not achieve similar success in HER2-negative diseases, including tumors with a low HER2 expression (immunohistochemistry (IHC)1+ or IHC2+/in situ hybridization (ISH)-negative) [5]. Novel anti-HER2 antibody–drug conjugates (ADCs), namely trastuzumab deruxtecan (T-DXd) and trastuzumab duocarmazine (SYD985), have demonstrated promising activity against breast cancer with low HER2 expression in early-phase clinical trials [6,7,8,9,10]. The DESTINY-Breast04 later confirmed T-DXd to be the new standard of care in pretreated metastatic breast cancer with low HER2 expression with remarkable improvement in both progression-free survival (PFS) and overall survival (OS) [11].

However, high-quality data are lacking on the clinical outcomes of breast cancers with HER2-low expression, particularly resectable disease [6,12]. Most previous studies were single-center or regional observational studies with conflicting results [13,14,15,16,17,18,19,20]. In addition, they may not represent the United States (US) patient population and failed to consider the possible interaction between treatment and clinical outcomes of HER2-low tumors [13,14,15,16,17,18,19,20,21]. Further, there is a knowledge gap on the prognostic role of HER2-low expression based on the genomic risk. The Oncotype Dx recurrence score (RS) is a 21-gene RS assay used in hormone receptor-positive (HR+) breast cancer to decide on the prognostic and predictive benefits of chemotherapy [22]. In node-positive early-stage HR+ HER2-negative breast cancer patients, dose-dense chemotherapy has consistently demonstrated benefits that vary based on the composite measure of patients’ recurrence risk [23]. In conjunction with various other clinicopathological risk factors predictive of recurrence, the inclusion of the genomic assay Oncotype DX RS can provide additional confidence to healthcare providers when making decisions about adjuvant chemotherapy for HR+ HER2-negative breast cancer patients. Research has indicated that the incorporation of Oncotype DX RS into clinical practice has effectively reduced discordance and subjectivity among practicing physicians regarding adjuvant chemotherapy recommendations [24]. Oncotype Dx RS was identified as a highly relevant prognostic factor for disease recurrence in early-stage HR+ HER2-negative breast cancer [25]. While previous studies have investigated the survival outcomes of HER2-low patients [26], none of them have specifically examined the association between HER2-low status and the Oncotype Dx RS. Therefore, this study examines and compares the clinical outcome of resectable HER2-low (IHC1+ or IHC2+/ISH-Negative) and HER2-zero (IHC 0) breast cancer and its prognostic association with the Oncotype Dx RS in HR+ breast cancer using the NCDB, a US national cancer outcome database.

## 2. Materials and Methods

### 2.1. Patient Selections

The NCDB, a joint program from the Commission on Cancer of the American College of Surgeons and the American Cancer Society, is a nationwide oncology outcomes database that collects information on approximately 70% of all new invasive cancer diagnoses in the US. 

**Figure 1:**
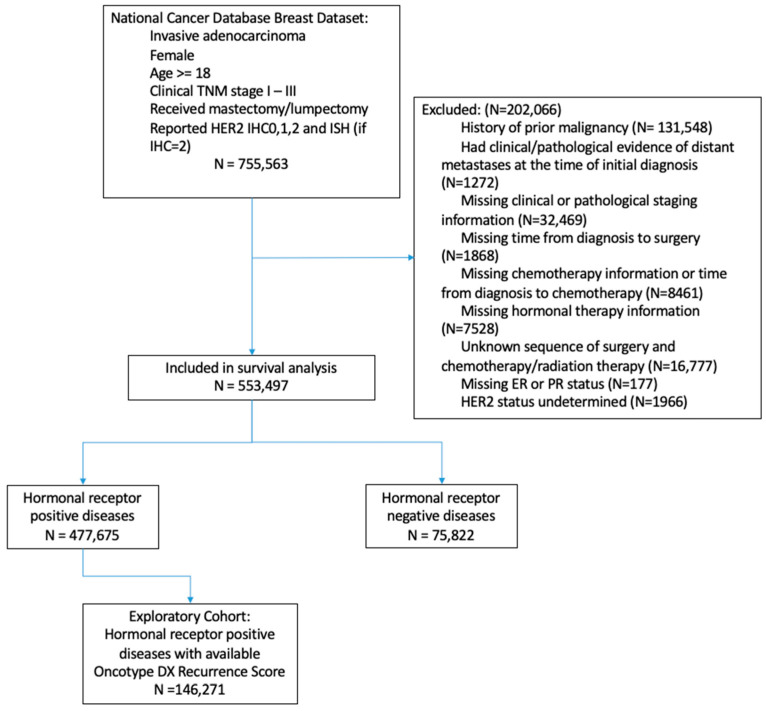
Study design. NCDB, National Cancer Database.

Between 1 January 2010 and 31 December 2017, 755,563 women newly diagnosed with breast cancer were identified. Women were included if they had invasive adenocarcinoma, clinical TNM stage 1–3 disease, complete information on age, race, hormonal receptor (HR) status (estrogen receptor-positive (ER+) or progesterone receptor-positive (PR+)), a zero/low expression level of HER2 (IHC score 0–2, and ISH status if IHC2+), and surgical resection (including lumpectomy or mastectomy). Exclusion criteria included history of prior malignancy, clinical/pathological evidence of distant metastases at the time of initial diagnosis, missing clinical/pathological staging information, missing time from diagnosis to surgery or to chemotherapy, missing chemotherapy/hormonal therapy information, unknown sequence of surgery and chemotherapy/radiation therapy, missing hormonal receptor status, and undetermined HER2 status (Figure 1).

Adjuvant chemotherapy (AC) was defined as chemotherapy within 90 days of surgery, and neoadjuvant chemotherapy (NAC) was defined as chemotherapy initiated ≥84 and ≤270 days before surgery [27,28]. The treatment coding in the NCDB is limited to the first course of treatment, defined as all treatments administrated before disease progression or recurrence. Oncotype Dx RS was extracted from Site-Specific Factor (SSF) 23. Similar to the TAILORx study, we defined patients with high-risk Oncotype Dx RS as those with an RS of ≥26 [22].

The level of HER2 expression was determined based on HER2 IHC and ISH summary results (SSF8, SSF11, SSF13) based on the 2018 American Society of Clinical Oncology/College of American Pathologists (ASCO/CAP) guidelines [4]. To be consistent with previous studies on HER2-low breast cancer, we used the terms HER2-zero (IHC0) and HER2-low (IHC 1+/IHC 2+ with negative ISH) in this study.

OS was the primary endpoint in all cohorts. In NAC patients, we also examined the pathological complete response (pCR, ypT0) rate as a secondary endpoint [29,30].

### 2.2. Statistical Analysis

Baseline characteristics between HER2-low and HER2-zero groups were compared using the *t*-test (for age) and Chi-square test, further stratified by HR status. Survival curves for OS were estimated using the Kaplan–Meier method. The log-rank test was used to compare OS across HER2 expression levels in HR+ and HR− patients who received chemotherapy (AC, NAC), hormonal therapy only (for HR+ cohort), and no systemic treatment. Similar analyses were performed in the HR-positive cohort with Oncotype Dx RS, and who received AC or hormonal therapy. We used the Chi-square test to compare the pCR rate between HER2-low and HER2-zero tumors in both HR-positive and HR-negative populations receiving NAC. Multivariable logistic regressions were also used to compare the odds ratios (OR) of pCR by HER2 expression level in both HR-positive and HR-negative populations. 

Three multivariable Cox regressions were performed for all HR-positive and HR-negative cohorts to estimate hazard ratios (HR), adjusting for age, race/ethnicity, household income, comorbidities, location, tumor grade, histology, hormonal receptor status (if applicable), clinical stage, lymph node involvement, the type of cancer center (where women received care), year of diagnosis, surgical resection, and systemic treatment type (any hormonal therapy, AC, or NAC). In subgroup analyses of all three cohorts, we performed the interaction between HER2 expression level and each of the important socio-demographic and clinical factors in separate multivariable Cox regression models, adjusting for the same covariates. We also performed an exploratory analysis in patients who had HR+ tumors with available Oncotype Dx RS and received AC and/or hormonal therapy. Similar models were used to compare the outcomes of the HER2-low and HER2-zero tumors, adjusting for previously mentioned covariates and Oncotype Dx RS as a continuous variable. 

To minimize misclassification bias from different reporting facilities, we performed the sensitivity analyses limited to the cases with initial diagnosis and all first-course treatment given at the same reporting facility. 

All statistical analyses were performed using SAS software, version 9.4 (SAS Institute Inc., Cary, NC, USA). All statistical significance testing was 2-sided at *p* < 0.05. Institutional review board review was exempted as the data were deidentified and publicly available upon request. Data were analyzed from 1 February 2021, through 2 February 2022.

## 3. Results

### 3.1. Patients Characteristics

**Figure 2:**
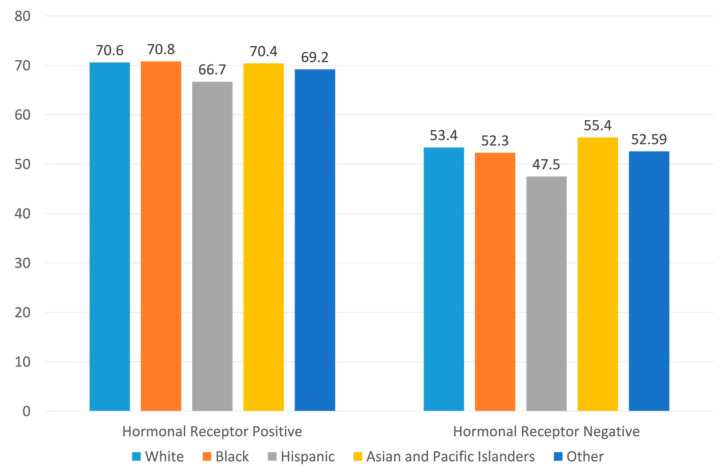
Prevalence of HER2-low tumors by patient race and tumor hormonal receptor status.

A total of 553,497 women met the inclusion criteria and were included in the final analysis; 177,298 (32.0%) women had HER2-zero breast cancers and 376,199 (68.0%) had HER2-low breast cancers. When stratified by hormonal receptor status, HER2-low tumors accounted for 336,147 of 447,675 (70.3%) cases in the HR+/HER2- population and 40,052 of 75,822 (52.8%) cases in the HR-/HER2- population. The prevalence of HER2-low tumors was similar across racial groups, with a slightly lower rate in the Hispanic population (Figure 2). Among all patients with available HER2 information, the median follow-up was 53.9 months (interquartile range (IQR): 35.1 months, 76.9 months).

HER2-low breast cancer patients tended to have public insurance at diagnosis (44.6% vs. 43.7%, *p* < 0.001). In terms of clinical risk factors, HER2-low disease was associated with positive HR status (89.4% vs. 79.8%, *p* < 0.001), well or moderately differentiated grade (well-differentiated: 26.5% vs. 23.8%; moderately differentiated: 46.1% vs. 42.1%, *p* < 0.001), ductal adenocarcinoma (76.1% vs. 72.4%, *p* < 0.001), and clinical stage I (64.5% vs. 63.2%, *p* < 0.001) but more lymph node involvement at diagnosis (no lymph node 69.7% vs. 71.0%, *p* < 0.001). Furthermore, patients with HER2-low breast cancers were more likely to be diagnosed in the early 2010s (*p* < 0.001) and to receive total mastectomy (37.0% vs. 36.3%, *p* < 0.001) and hormonal treatment (80.1% vs. 70.9%, *p* < 0.001) but were less likely to receive neoadjuvant and adjuvant chemotherapy (NAC: 8.7% vs. 10.8%; AC: 23.8% vs. 25.4%, both *p* < 0.001) (Table 1).

When stratified by tumor hormonal status, most socio-demographic and clinical factors were overall balanced between HER2-low and HER2-zero tumors (Table S1A,B). In the exploratory cohort with HR-positive tumors and Oncotype Dx RS results, the distribution of RS was largely comparable between HER2-low and HER2-zero tumors, despite the RS of HER2-low tumors being slightly more likely to be 16–25 (36.9% vs. 36. 7%) compared to HER2-zero, where the RS was slightly more likely to be 0–15 (50.6% vs. 49.9%) or 26–100 (13.3% vs. 13.2%, all *p*= 0.008) (Table 2).

### 3.2. Kaplan–Meier Estimates

**Figure 3:**
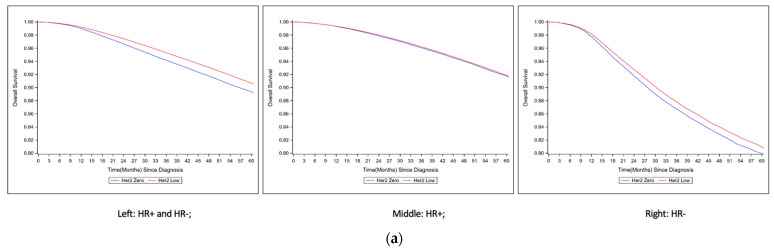

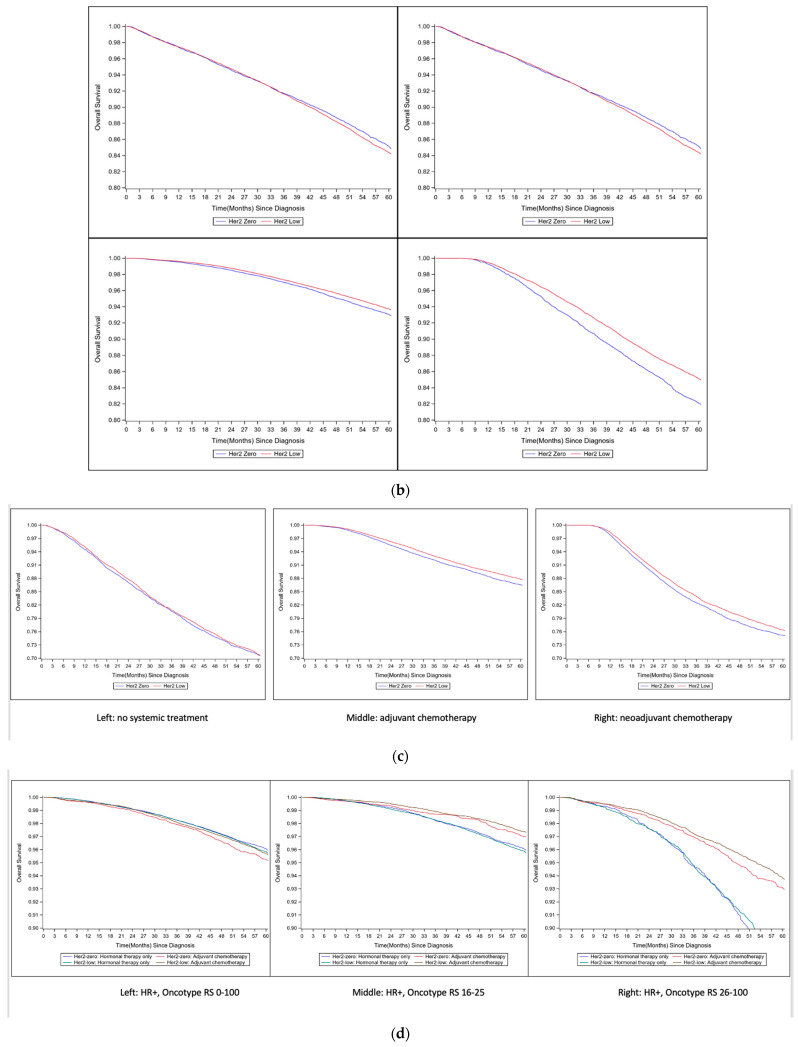
(**a**) Survival curve by level of HER2 expression and hormone receptor status. (**b**) Survival curve by level of HER2 expression and type of treatment for HR+ breast cancer. Upper left: no systemic treatment; upper right: adjuvant hormonal therapy only; lower left: adjuvant chemotherapy; lower right: neoadjuvant chemotherapy. (**c**) Survival curve by level of HER2 expression and type of treatment for HR- breast cancer. (**d**) Survival curve by level of HER2 expression and type of treatment for HR+ breast cancer with Oncotype DX recurrence scores.

After a median follow-up of 53.9 months, women with HER2-low breast cancer had higher 5-year OS rates than those with HER2-zero breast cancer (92.9% vs. 92.0%; log-rank *p* < 0.001). In fact, we observed a trend toward better survival of HER2 2+/ISH negative tumors compared to HER2 1+ and HER2 0 tumors (IHC 2+: 93.0% vs. IHC1+: 92.9% vs. IHC0: 92.0%; log-rank *p* < 0.001). In stratified analyses by HR status, HR+/HER2-low and HR+/HER2-zero tumors had similar 5-year OS in HR+ groups (log-rank *p* = 0.38), whereas HR-/HER2-low tumors had higher 5-year OS than HR-/HER2-zero tumors (log-rank *p* < 0.001) (Figure 3a and Figure S1a). However, this survival advantage among HER2-low tumors was observed in women who received adjuvant or neoadjuvant chemotherapy but not among those who received no systemic therapy or only hormonal therapy (Figure 3b,c and Figure S1b,c). Similar results were observed in the exploratory cohort stratified by Oncotype Dx RS (Figure 3d). The survival difference was more pronounced in patients with Oncotype Dx RSs of 16–25 and 26–100, with the highest difference observed in the Oncotype Dx RS 26–100 group. Among women with higher Oncotype Dx RS results (26–100) treated with chemotherapy, those with HER2-low breast cancer had higher 5-year OS than HER2-zero breast cancer.

### 3.3. Response to Neoadjuvant Chemotherapy

**Figure 4:**
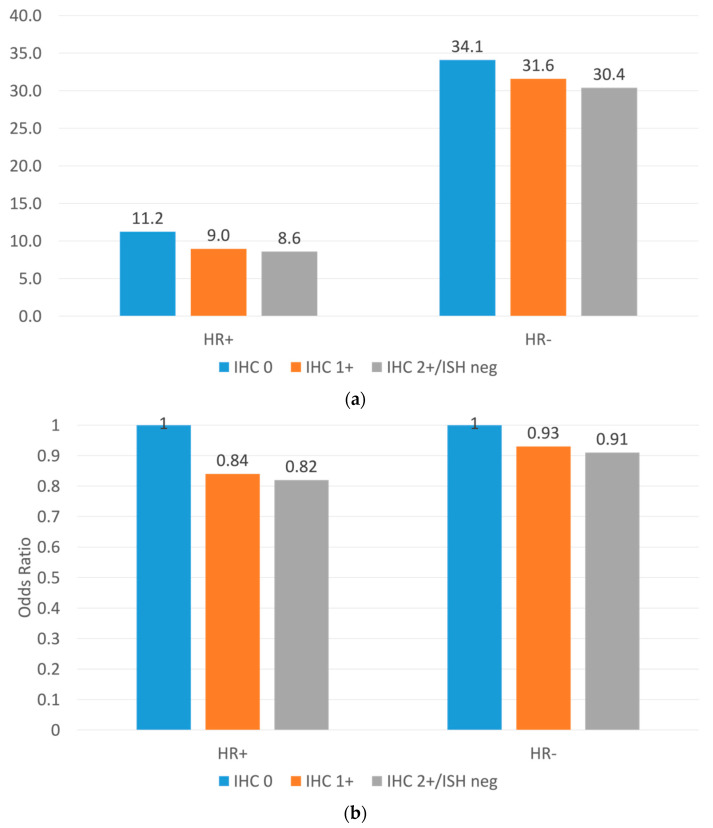
(**a**) Unadjusted pathological complete response (PCR) rate (%) after neoadjuvant chemotherapy, by HER2 IHC score and hormonal receptor status. (**b**) Adjusted odds ratio (OR) of pathological complete response (PCR) after neoadjuvant chemotherapy, by HER2 IHC score and hormonal receptor status. Multivariable logistic regression models were adjusted for age, race/ethnicity, household income, comorbidities, location, tumor grade, histology, lymph node involvement, and the type of cancer center (where women received care).

HER2 2+/ISH-negative and HER2 1+ tumors had a lower pCR rate than HER2-zero tumors among both HR-positive and HR-negative diseases (HR+: 8.6% vs. 9.0% vs. 11.2%, *p* < 0.001; HR−: 30.4% vs. 31.6% vs. 34.1%, *p* < 0.001) (Figure 4a). The association between HER2 expression level and pCR rates remains significant in the adjusted analyses (both *p* < 0.001) (Figure 4b).

### 3.4. Multivariable Survival Analyses

After adjusting for socio-demographic and clinical factors, HER2-low expression remained associated with slightly better survival (HR 0.94, 95% CI (0.92–0.95), *p* < 0.001). The survival advantage was even more evident in the HR-negative cohort (HR 0.87, 95% CI (0.84–0.90), *p* < 0.001) than in the HR-positive cohort (HR 0.97, 95% CI (0.95–0.99), *p* = 0.01). In addition, HER2 2+/ISH-negative breast cancer had a marginal but significant survival advantage compared to HER2 1+ breast cancer (HER2 2+/ISH negative vs. HER2 1+, HR 0.95, 95% CI (0.93–0.98), *p* < 0.001).

**Figure 5:**
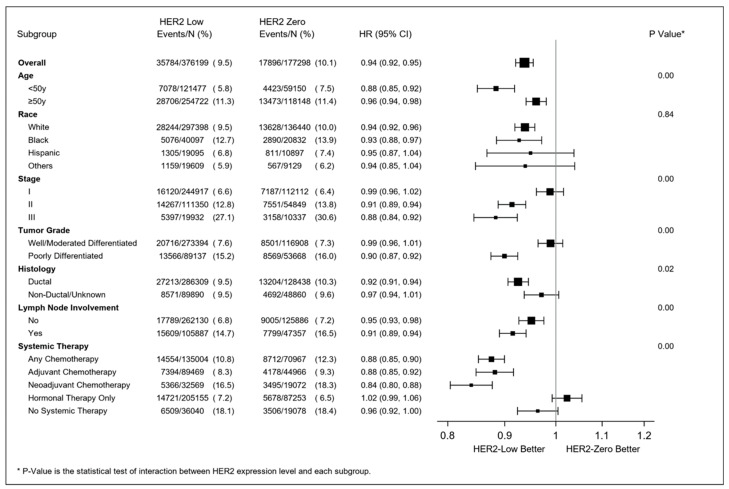
Adjusted hazard ratio (HER2-low vs. HER2-zero breast cancer) in resectable breast cancer. The Cox regression models were adjusted for age, race/ethnicity, household income, comorbidities, location, tumor grade, stage, histology, hormonal receptor status, lymph node involvement at diagnosis, the type of cancer center (where women received care), year of diagnosis, and treatment type. Adjuvant chemotherapy (AC) was strictly defined as chemotherapy within 90 days of surgery, and neoadjuvant chemotherapy (NAC) was strictly defined as chemotherapy initiated ≥84 and ≤270 days before surgery. Therefore, the sum of the numbers of patients who received adjuvant chemotherapy and neoadjuvant chemotherapy may be less than the number of patients who received any chemotherapy.

Further subgroup analyses showed that the survival advantage of HER2-low tumors was significantly correlated with younger age (<50 year), later clinical stage, higher tumor grade, and chemotherapy receipt (all p-interaction < 0.01). This advantage was not associated with race (p-interaction = 0.84) or lymph node involvement at diagnosis (p-interaction = 0.11) (Figure 5).

**Figure 6:**
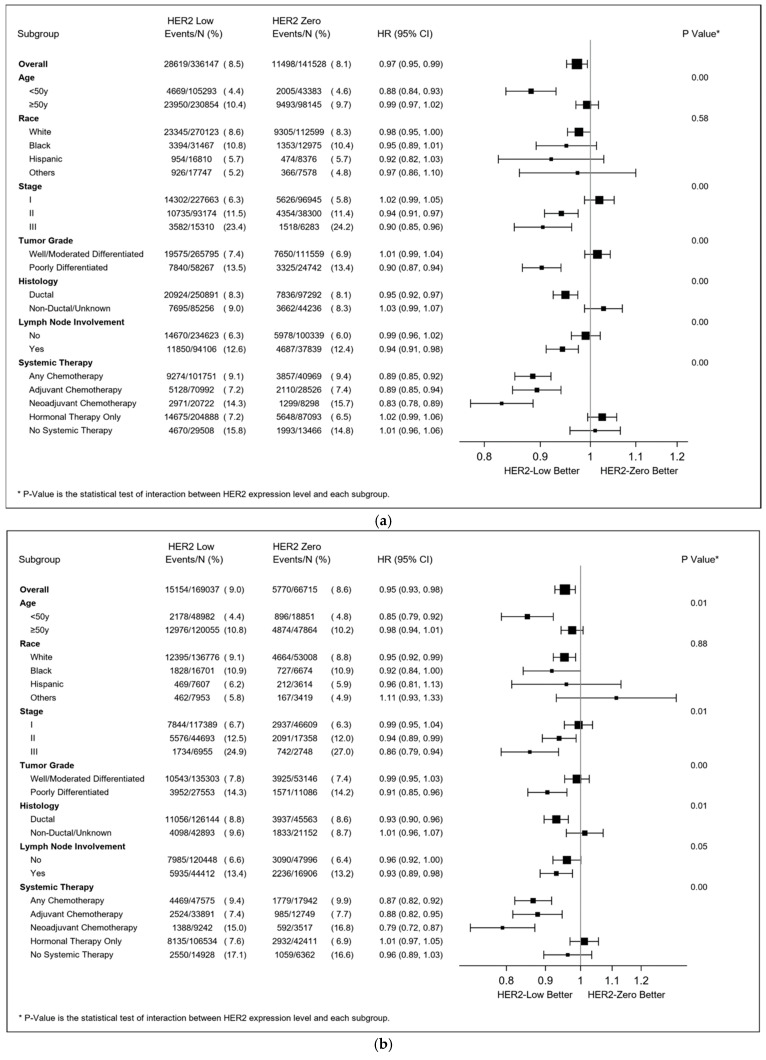

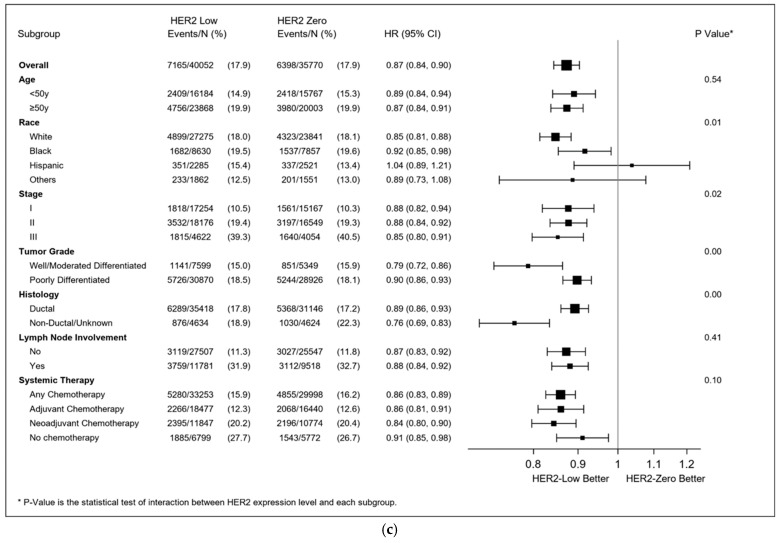
(**a**) Adjusted hazard ratio (HER2-low vs. HER2-zero breast cancer) in resectable HR+ breast cancer. The Cox regression models were adjusted for age, race/ethnicity, household income, comorbidities, location, tumor grade, stage, histology, lymph node involvement at diagnosis, the type of cancer center (where women received care), year of diagnosis, and treatment type. Adjuvant chemotherapy (AC) was strictly defined as chemotherapy within 90 days of surgery, and neoadjuvant chemotherapy (NAC) was strictly defined as chemotherapy initiated ≥84 and ≤270 days before surgery. Therefore, the sum of the numbers of patients who received adjuvant chemotherapy and neoadjuvant chemotherapy may be less than the number of patients who received any chemotherapy. (**b**) Adjusted hazard ratio (HER2-low vs. HER2-zero breast cancer) in resectable HR+ breast cancer with available Oncotype RSs. The Cox regression models were adjusted for age, race/ethnicity, household income, comorbidities, location, tumor grade, stage, histology, lymph node involvement at diagnosis, the type of cancer center (where women received care), year of diagnosis, treatment type, and Oncotype RS. Adjuvant chemotherapy (AC) was strictly defined as chemotherapy within 90 days of surgery, and neoadjuvant chemotherapy (NAC) was strictly defined as chemotherapy initiated ≥84 and ≤270 days before surgery. Therefore, the sum of the numbers of patients who received adjuvant chemotherapy and neoadjuvant chemotherapy may be less than the number of patients who received any chemotherapy. (**c**) Adjusted hazard ratio (HER2-low vs. HER2-zero breast cancer) in resectable HR- breast cancer. The Cox regression models were adjusted for age, race/ethnicity, household income, comorbidities, location, tumor grade, stage, histology, lymph node involvement at diagnosis, the type of cancer center (where women received care), year of diagnosis, and treatment type. Adjuvant chemotherapy (AC) was strictly defined as chemotherapy within 90 days of surgery, and neoadjuvant chemotherapy (NAC) was strictly defined as chemotherapy initiated ≥84 and ≤270 days before surgery. Therefore, the sum of the numbers of patients who received adjuvant chemotherapy and neoadjuvant chemotherapy may be less than the number of patients who received any chemotherapy.

In the HR-positive population, the survival advantage of HER2-low tumors was more significant among women who were aged <50 y who had ductal adenocarcinoma and poorly differentiated tumors and those who received any chemotherapy (including AC and NAC) but not hormonal therapy (all p-interaction < 0.01) (Figure 6a). In the exploratory cohort of HR-positive tumors with available Oncotype Dx RSs, this survival advantage was observed primarily in patients who received AC (HR 0.87 95% CI (0.77–0.99), p-interaction = 0.01), with adjustment for Oncotype Dx RS (Figure 6b). In the HR-negative cohort, the advantage was more significant among patients with well/moderately differentiated tumors and non-ductal carcinoma (all p-interaction < 0.01) (Figure 6c).

When limited to the cases with initial diagnosis and all first-course treatments given at the same reporting facility, our sensitivity analyses showed largely similar results (Figures S2 and S3a,b).

## 4. Discussion

This study found that approximately 70% of HR+/HER2- resectable breast cancers and 50% of HR-/HER2- resectable breast cancers had low HER2 expression, with slight variation across racial groups. HER2-low breast cancers had longer survival than HER2-zero breast cancers, regardless of their HR status. In the HR-positive cohort, the survival advantage was primarily observed among women who received adjuvant or neoadjuvant chemotherapy. This survival advantage remained significant even after adjusting for the Oncotype Dx RS.

Our study is a comprehensive study on the epidemiology and outcomes of resectable HER2-low breast cancer using a US national dataset. Furthermore, this is the first study describing the prognostic impact of HER2-low expression in HR+ breast cancer patients in accordance with the Oncotype Dx RS using a large national dataset. We also reported detailed data on Black and Hispanic patients with resectable HER2-low breast cancer. We found that the prevalence and survival advantage of resectable HER2-low tumors were similar across racial groups. In addition, we observed that the survival advantage of HR+/HER2-low tumors was more significant among patients younger than 50 years with poorly differentiated tumors and ductal adenocarcinoma. Future research is warranted to explore any biological difference in HER2-low tumors across various subgroups.

Our study suggested that HER2-low expression was more likely to be HR-positive with invasive ductal adenocarcinoma, consistent with most previous studies [14,15,17,18,21,31,32,33]. Our results further showed that HER2-low breast cancers had more nodal involvement but lower tumor grade at diagnosis. A secondary analysis of four European neoadjuvant trials supported this finding, and a single-center US study also observed lower tumor grade in HER2-low tumors [21,31,32]. Interestingly, Eggimann et al. found that early-stage HER2-low tumors had more lymph node involvement but higher tumor grade and Ki-67 index than HER2-zero tumors based on data from the German cancer registries [15]. Other studies also reported conflicting pathological characteristics by HR status [14,16,18]. One possibility for these inconsistent clinical and molecular characteristics could be genetic heterogeneity or varying standards of pathological reports by countries. While the NCDB does not have detailed genomic or sequencing data, our exploratory analyses suggested no clear difference in Oncotype Dx RS between HER2-low and HER2-zero tumors.

Interestingly, the survival advantage of HER2-low breast cancer seems associated with the receipt of chemotherapy (AC or NAC). We did not observe a similar survival advantage in those who received only hormonal therapy or no systemic therapy. No previous study has examined the clinical outcomes of resectable HER2-low breast cancer by systemic treatment. Considering that low-grade tumors tend to be less proliferative and sensitive to chemotherapy [34,35], it is surprising to observe the survival benefit of HER2-low tumors only in patients who received chemotherapy but not in patients with other or no treatment. Our exploratory analyses further suggested that this interaction of chemotherapy with the HER2 subtype remained significant even after adjusting for Oncotype DX RS. This result may indicate that the current 21-gene genomic signature might not fully explain the difference in response to chemotherapy between HER2-low and HER2-zero tumors. Nonetheless, as the NCDB provides no further details on treatment, future studies are warranted to confirm this interaction between chemotherapy and HER2-low tumors in another cohort and explore its potential mechanism.

In the exploratory analyses adjusting for Oncotype Dx RS, we did not observe any significant difference in OS by HER2 expression level in most subgroups, which was partially inconsistent with results from the HR+ populations. A possible explanation is that Oncotype Dx RSs were mainly ordered for patients with stage I/II cancer to avoid chemotherapy, thus, this cohort included limited patients who received chemotherapy. Oncotype Dx RS might still partially account for survival differences, despite similar Oncotype Dx RSs between HER2-low and HER2-zero tumors. It is also observed that among patients who received AC in the group with high Oncotype Dx RS, those with HER2-low breast cancer had improved survival compared to those with HER2-zero breast cancer. A retrospective single institutional study by Mutai et al. also showed similar findings [36]. However, further research is needed to examine the interaction between Oncotype Dx RS and HER2 expression level and its impact on relapse or survival.

In patients who received NAC, HER2-low expression was associated with lower pCR rates but more prolonged OS. Our results confirmed the findings of a recent pooled analysis of four neoadjuvant trial data. Denkert et al. found that HER2-low tumors had significantly lower pCR rates (statistically significant in the HR+ tumors but not significant in the HR− tumors) and longer disease-free survival/OS (statistically significant in the HR− tumors but not significant in the HR+ tumors). In a single-institute retrospective study, Tarantino et al. reported that the lower pCR observed with the HER2-low expression in those who received NAC was due to the positive association with the level of ER expression [21]. In contrast to our results, they noted that after a follow-up of 10 months, there were no significant survival differences between HER2-low and HER2-zero early-stage breast cancer when stratified based on HR status and after adjusting for the confounders [37]. Inadequate sample sizes may explain the insignificant results, as the hazard ratios were similar to our results but with larger margins. With a much larger sample size, longer follow-up, and higher statistical power compared to the above-mentioned studies, our study provided more robust estimates and identified the difference in pCR rates and OS between HER2-low and HER2-zero tumors. However, it is important to note that the survival difference between the two groups was marginal, and the statistical difference may not always reflect clinical benefit.

The major strength of this study is that the NCDB fully reflects the diversity of breast cancer patients and treatment paradigms in the US [38,39]. With a considerably more extensive and more diverse sample, we examined the difference between HER2-low and HER2-zero tumors in most subgroups with adequate statistical power. Another strength of this study is that we examined the survival of HER2-low tumors under different treatments and in association with the genomic risk scoring, which has previously been reported only as a single-center experience. We also performed sensitivity analyses to minimize misclassification bias from different reporting facilities, which is one of the great strengths of the study. Admittedly, this is only a hypothesis-generating study due to the observational nature and the limitations of the NCDB dataset. Further validations are warranted in other real-world outcome datasets with more clinical and treatment information.

This study has several limitations. First, the NCDB is based on hospital reports rather than data from populational registries. This study should not be interpreted as a nationally representative study despite its large and diverse sample since the data are mostly hospital-based as opposed to general population-based data, and, therefore, generalizability may be limited [39]. However, given the results obtained from a large sample, the data can be utilized for research purposes, especially for understanding the treatment patterns for HER2-low disease in different institutions. Due to the observational nature of the NCDB, the HER2 IHC and ISH results were based on reports from cancer centers and were not confirmed in a central pathology laboratory, which is needed, as in most cases there can be disparities in the IHC reports provided by pathologists [40]. As the ASCO/CAP revised the definition of HER2 positivity (the elimination of the ISH equivocal category and a change in the IHC 2+ cut-off from 30% to 10% stained cells) during and after our study period [4,41], our study may have a misclassification bias, which potentially could lead to a minor overestimation of the survival difference between HER2 IHC2+/1+ and HER2 IHC0 tumors. In addition, HER2 itself is a dynamic entity and it can vary over a period of time even within the same tumor site or within different organ sites biopsied at the same time from the same patient [12]. This might have affected the accuracy and consistency of the reported HER2 values and thereby could have affected the validity of some of the results of the study, as HER2 is a crucial variable for our study. However, biases that can occur due to the lack of a central reporting system occur in most of the HER2 studies involving a retrospective study design. In addition, when patients receive systemic treatments outside the reporting facility, their chemotherapy or hormonal therapy information may be missing or misclassified as “no chemotherapy” or “no hormonal therapy”. However, our sensitivity analyses suggested results consistent with the main findings (Table S2). Further, the NCDB only recorded the treatment information from the first course, and it did not report data regarding the exact chemotherapy regimen, the number of cycles administered, and treatments at relapse. Since further treatments after first-line have implications in terms of prognosis, the extrapolation of the clinical findings from the NCDB database (where this information is missing) to the general population should be carried out with caution. In addition, we could not estimate the HER2-low tumors’ response to adjuvant CDK4/6 inhibitors or neoadjuvant immune-checkpoint inhibitors, as both drugs were not available during the study period. Given the NCDB does not capture genetic information such as BRCA ½ or molecular data such as the KI-67 index, we could not assess the potential correlation of these factors with the HER2 status and clinical outcomes. Lastly, the retrospective nature of this study might have introduced selection bias and incomplete information and could have predisposed the results to other unknown confounders, which we cannot adjust for with statistical models. These limitations might have impacted the internal validity of the study, potentially affecting the interpretation of some of the observed associations.

Our findings warrant further validation, more translational research on HER2-low tumor biology, and the clinical development of novel ADCs in patients with high-risk resectable diseases. Further studies focusing on HER2-low tumors would provide valuable insights for the development of personalized treatments and enable the potential de-escalation of systemic therapy while ensuring that treatment efficacy remains unaffected [42]. Since our study primarily concentrated on invasive breast cancer, we have not examined the correlation between HER2-low expression in ductal carcinoma in situ (DCIS) and lobular carcinoma in situ (LCIS). Given that research indicates a potential link between HER2-positive DCIS and elevated breast cancer recurrence rates, along with adverse clinicopathological characteristics [43], further investigation into the significance of HER2-low expression in both DCIS and LCIS is warranted for future studies. Further, these findings may have clinical implications for other tumor types like lung and gastric carcinoma with low HER2 expression.

## 5. Conclusions

HER2-low expression was associated with moderately better survival in patients with resectable breast cancers, irrespective of their HR status. The survival advantage was mainly observed in patients who received chemotherapy. Similar findings were observed in HR+ breast cancer patients with high Oncotype Dx RSs. Among patients who received adjuvant chemotherapy with a high Oncotype Dx RS (26–100), those with HER2-low tumors had higher survival. Further research is warranted to confirm this finding and explore its underlying mechanism. New treatment strategies based on novel anti-HER2 therapies should be investigated for high-risk resectable HER2-low breast cancer.

## Figures and Tables

**Table 1:** Characteristics of patients with HER2-low and HER2-zero resectable breast cancer.

Variable	Level	N	Overall N = 553,497	Her2-Low N = 376,199	Her2-Zero N = 177,298	*p*-Value
Age		553,497	60.6 ± 12.6	60.7 ± 12.6	60.4 ± 12.7	<0.001
Race	1. White	553,497	433,838 (78.4%)	297,398 (79.1%)	136,440 (77.0%)	<0.001
	2. Black		60,929 (11.0%)	40,097 (10.7%)	20,832 (11.7%)	
	3. Hispanic		29,992 (5.4%)	19,095 (5.1%)	10,897 (6.1%)	
	4. Asian and Pacific Islanders		20,555 (3.7%)	14,123 (3.8%)	6432 (3.6%)	
	5. Other or unknown		8183 (1.5%)	5486 (1.5%)	2697 (1.5%)	
Insurance	1. Private	55,3497	294,343 (53.2%)	199,250 (53.0%)	95,093 (53.6%)	<0.001
	2. Public insurance		245,077 (44.3%)	167,671 (44.6%)	77,406 (43.7%)	
	3. Uninsured		8952 (1.6%)	5889 (1.6%)	3063 (1.7%)	
	4. Unknown		5125 (0.9%)	3389 (0.9%)	1736 (1.0%)	
Household Income	1. <$40,227	553,497	70,895 (12.8%)	47,742 (12.7%)	23,153 (13.1%)	<0.001
	2. $40,227–$50,353		94,587 (17.1%)	64,914 (17.3%)	29,673 (16.7%)	
	3. $50,354–$63,332		111,351 (20.1%)	75,856 (20.2%)	35,495 (20.0%)	
	4. ≥$63,333		200,876 (36.3%)	133,969 (35.6%)	66,907 (37.7%)	
	5. Unknown		75,788 (13.7%)	53,718 (14.3%)	22,070 (12.4%)	
Treatment Setting	1. Community cancer program	553,497	36,308 (6.6%)	25,402 (6.8%)	10,906 (6.2%)	<0.001
	2. Comprehensive community cancer program		219,471 (39.7%)	153,811 (40.9%)	65,660 (37.0%)	
	3. Academic comprehensive cancer program		159,912 (28.9%)	102,545 (27.3%)	57,367 (32.4%)	
	4. Integrated network cancer program		112,286 (20.3%)	77,899 (20.7%)	34,387 (19.4%)	
	5. Unknown		25,520 (4.6%)	16,542 (4.4%)	8978 (5.1%)	
Treatment Location	1. Metro	553,497	470,060 (84.9%)	317,798 (84.5%)	152,262 (85.9%)	<0.001
	2. Urban		62,460 (11.3%)	44,003 (11.7%)	18,457 (10.4%)	
	3. Rural		8055 (1.5%)	5826 (1.5%)	2229 (1.3%)	
	4. Unknown		12,922 (2.3%)	8572 (2.3%)	4350 (2.5%)	
Histology	1. Ductal adenocarcinoma	553,497	414,747 (74.9%)	286,309 (76.1%)	128,438 (72.4%)	<0.001
	2. Lobular adenocarcinoma		58,463 (10.6%)	38,155 (10.1%)	20,308 (11.5%)	
	3. Mixed or unknown histology		80,287 (14.5%)	51,735 (13.8%)	28,552 (16.1%)	
Tumor Grade	1. Well differentiated	553,497	142,090 (25.7%)	99,817 (26.5%)	42,273 (23.8%)	<0.001
	2. Moderately differentiated		248,212 (44.8%)	173,577 (46.1%)	74,635 (42.1%)	
	3. Poorly differentiated/undifferentiated		142,805 (25.8%)	89,137 (23.7%)	53,668 (30.3%)	
	4. Unknown		20,390 (3.7%)	13,668 (3.6%)	6722 (3.8%)	
Clinical Stage	Stage I	553,497	357,029 (64.5%)	244,917 (65.1%)	112,112 (63.2%)	<0.001
	Stage II		166,199 (30.0%)	111,350 (29.6%)	54,849 (30.9%)	
	Stage III		30,269 (5.5%)	19,932 (5.3%)	10,337 (5.8%)	
Lymph Node Involvement at Diagnosis	1. No lymph node	553,497	388,016 (70.1%)	262,130 (69.7%)	125,886 (71.0%)	<0.001
	2. 1–3 lymph nodes		113,966 (20.6%)	78,894 (21.0%)	35,072 (19.8%)	
	3. 4+ lymph nodes		39,278 (7.1%)	26,993 (7.2%)	12,285 (6.9%)	
	4. Unknown		12,237 (2.2%)	8182 (2.2%)	4055 (2.3%)	
Hormonal Receptor Status	1. Yes	553,497	477,675 (86.3%)	336,147 (89.4%)	141,528 (79.8%)	<0.001
	2. No		75,822 (13.7%)	40,052 (10.6%)	35,770 (20.2%)	
Surgical Treatment	1. Lumpectomy or partial mastectomy	553,497	349,935 (63.2%)	236,913 (63.0%)	113,022 (63.7%)	<0.001
	2. Total mastectomy		203,562 (36.8%)	139,286 (37.0%)	64,276 (36.3%)	
Adjuvant Radiation	1. Yes	553,497	368,206 (66.5%)	250,112 (66.5%)	118,094 (66.6%)	0.363
	2. No		185,291 (33.5%)	126,087 (33.5%)	59,204 (33.4%)	
Chemotherapy	1. Yes	553,497	205,971 (37.2%)	135,004 (35.9%)	70,967 (40.0%)	<0.001
	2. No		347,526 (62.8%)	241,195 (64.1%)	106,331 (60.0%)	
Neoadjuvant Chemotherapy	1. Yes	553,497	51,641 (9.3%)	32,569 (8.7%)	19,072 (10.8%)	<0.001
	2. No		501,856 (90.7%)	343,630 (91.3%)	158,226 (89.2%)	
Adjuvant Chemotherapy	1. Yes	553,497	134,435 (24.3%)	89,469 (23.8%)	44,966 (25.4%)	<0.001
	2. No		419,062 (75.7%)	286,730 (76.2%)	132,332 (74.6%)	
Hormone Treatment	1. Yes	553,497	426,986 (77.1%)	301,202 (80.1%)	125,784 (70.9%)	<0.001
	2. No		126,511 (22.9%)	74,997 (19.9%)	51,514 (29.1%)	
Comorbidity Score	0	553,497	459,789 (83.1%)	312,377 (83.0%)	147,412 (83.1%)	0.022
	1		72,834 (13.2%)	49,709 (13.2%)	23,125 (13.0%)	
	2		14,976 (2.7%)	10,197 (2.7%)	4779 (2.7%)	
	≥3		5898 (1.1%)	3916 (1.0%)	1982 (1.1%)	
Year of Diagnosis	2010	553,497	48,104 (8.7%)	33,414 (8.9%)	14,690 (8.3%)	<0.001
	2011		55,715 (10.1%)	38,793 (10.3%)	16,922 (9.5%)	
	2012		60,516 (10.9%)	43,645 (11.6%)	16,871 (9.5%)	
	2013		68,550 (12.4%)	47,071 (12.5%)	21,479 (12.1%)	
	2014		72,624 (13.1%)	50,208 (13.3%)	22,416 (12.6%)	
	2015		78,184 (14.1%)	53,022 (14.1%)	25,162 (14.2%)	
	2016		83,765 (15.1%)	55,478 (14.7%)	28,287 (16.0%)	
	2017		86,039 (15.5%)	54,568 (14.5%)	31,471 (17.8%)	

**Table 2:** Characteristics of patients with HER2-low and HER2-zero HR+ resectable breast cancer with available Oncotype DX recurrence scores.

Variable	Level	N	Overall N = 146,271	Her2-Low N = 104,517	Her2-Zero N = 41,754	*p*-Value
Oncotype Dx Recurrence Score	0–15	146,271	73,250 (50.1%)	52,114 (49.9%)	21,136 (50.6%)	0.008
	16–25		53,649 (36.7%)	38,592 (36.9%)	15,057 (36.1%)	
	26–100		19,372 (13.2%)	13,811 (13.2%)	5561 (13.3%)	
Age		146,271	58.8 ± 10.4	58.7 ± 10.5	58.9 ± 10.4	0.004
Race	1. White	146,271	120,210 (82.2%)	86,244 (82.5%)	33,966 (81.3%)	<0.001
	2. Black		11,471 (7.8%)	8257 (7.9%)	3214 (7.7%)	
	3. Hispanic		6726 (4.6%)	4491 (4.3%)	2235 (5.4%)	
	4. Asian and Pacific Islanders		5687 (3.9%)	4011 (3.8%)	1676 (4.0%)	
	5. Other or unknown		2177 (1.5%)	1514 (1.4%)	663 (1.6%)	
Insurance	1. Private	146,271	89,946 (61.5%)	64,140 (61.4%)	25,806 (61.8%)	0.148
	2. Public insurance		53,452 (36.5%)	38,352 (36.7%)	15,100 (36.2%)	
	3. Uninsured		1627 (1.1%)	1136 (1.1%)	491 (1.2%)	
	4. Unknown		1246 (0.9%)	889 (0.9%)	357 (0.9%)	
Household Income	1. <$40,227	146,271	15,136 (10.3%)	10,839 (10.4%)	4297 (10.3%)	<0.001
	2. $40,227–$50,353		22,687 (15.5%)	16,528 (15.8%)	6159 (14.8%)	
	3. $50,354–$63,332		29,330 (20.1%)	21,036 (20.1%)	8294 (19.9%)	
	4. ≥$63,333		57,928 (39.6%)	40,237 (38.5%)	17,691 (42.4%)	
	5. Unknown		21,190 (14.5%)	15,877 (15.2%)	5313 (12.7%)	
Treatment Setting	1. Community cancer program	146,271	8246 (5.6%)	6072 (5.8%)	2174 (5.2%)	<0.001
	2. Comprehensive community cancer program		57,187 (39.1%)	42,054 (40.2%)	15,133 (36.2%)	
	3. Academic comprehensive cancer program		46,672 (31.9%)	31,274 (29.9%)	15,398 (36.9%)	
	4. Integrated network cancer program		29,872 (20.4%)	21,975 (21.0%)	7897 (18.9%)	
	5. Unknown		4294 (2.9%)	3142 (3.0%)	1152 (2.8%)	
Treatment Location	1. Metro	146,271	124,162 (84.9%)	88,356 (84.5%)	35,806 (85.8%)	<0.001
	2. Urban		16,165 (11.1%)	12,019 (11.5%)	4146 (9.9%)	
	3. Rural		2076 (1.4%)	1558 (1.5%)	518 (1.2%)	
	4. Unknown		3868 (2.6%)	2584 (2.5%)	1284 (3.1%)	
Histology	1. Ductal adenocarcinoma	146,271	107,552 (73.5%)	78,570 (75.2%)	28,982 (69.4%)	<0.001
	2. Lobular adenocarcinoma		18,336 (12.5%)	12,042 (11.5%)	6294 (15.1%)	
	3. Mixed or unknown histology		20,383 (13.9%)	13,905 (13.3%)	6478 (15.5%)	
Tumor Grade	1. Well differentiated	146,271	41,082 (28.1%)	29,755 (28.5%)	11,327 (27.1%)	<0.001
	2. Moderately differentiated		79,377 (54.3%)	56,355 (53.9%)	23,022 (55.1%)	
	3. Poorly differentiated/undifferentiated		21,597 (14.8%)	15,359 (14.7%)	6238 (14.9%)	
	4. Unknown		4215 (2.9%)	3048 (2.9%)	1167 (2.8%)	
Clinical Stage	Stage I	146,271	111,871 (76.5%)	80,023 (76.6%)	31,848 (76.3%)	0.282
	Stage II		33,795 (23.1%)	24,075 (23.0%)	9720 (23.3%)	
	Stage III		605 (0.4%)	419 (0.4%)	186 (0.4%)	
Lymph Node Involvement	1. No lymph node	146,271	120,058 (82.1%)	85,804 (82.1%)	34,254 (82.0%)	0.622
	2. 1–3 lymph nodes		24,643 (16.8%)	17,612 (16.9%)	7031 (16.8%)	
	3. 4+ lymph nodes		912 (0.6%)	634 (0.6%)	278 (0.7%)	
	4. Unknown		658 (0.4%)	467 (0.4%)	191 (0.5%)	
Hormonal Receptor Status	1. Yes	146,271	146,271 (100.0%)	104,517 (100.0%)	41,754 (100.0%)	-
Surgical Treatment	1. Lumpectomy or partial mastectomy	146,271	102,047 (69.8%)	72,410 (69.3%)	29,637 (71.0%)	<0.001
	2. Total mastectomy		44,224 (30.2%)	32,107 (30.7%)	12,117 (29.0%)	
Adjuvant Radiation	1. Yes	146,271	102,105 (69.8%)	72,605 (69.5%)	29,500 (70.7%)	<0.001
	2. No		44,166 (30.2%)	31,912 (30.5%)	12,254 (29.3%)	
Adjuvant Chemotherapy	1. Yes	146,271	28,879 (19.7%)	20,726 (19.8%)	8153 (19.5%)	0.187
	2. No		117,392 (80.3%)	83,791 (80.2%)	33,601 (80.5%)	
Hormone Treatment	1. Yes	146,271	144,993 (99.1%)	103,637 (99.2%)	41,356 (99.0%)	0.039
	2. No		1278 (0.9%)	880 (0.8%)	398 (1.0%)	
Comorbidity Score	0	146,271	123,996 (84.8%)	88,494 (84.7%)	35,502 (85.0%)	0.113
	1		17,937 (12.3%)	12,927 (12.4%)	5010 (12.0%)	
	2		3245 (2.2%)	2335 (2.2%)	910 (2.2%)	
	≥3		1093 (0.7%)	761 (0.7%)	332 (0.8%)	
Year of Diagnosis	2010	146,271	8747 (6.0%)	6360 (6.1%)	2387 (5.7%)	<0.001
	2011		11,556 (7.9%)	8475 (8.1%)	3081 (7.4%)	
	2012		13,880 (9.5%)	10,541 (10.1%)	3339 (8.0%)	
	2013		17,042 (11.7%)	12,449 (11.9%)	4593 (11.0%)	
	2014		19,750 (13.5%)	14,303 (13.7%)	5447 (13.0%)	
	2015		22,835 (15.6%)	16,392 (15.7%)	6443 (15.4%)	
	2016		25,392 (17.4%)	17,748 (17.0%)	7644 (18.3%)	
	2017		27,069 (18.5%)	18,249 (17.5%)	8820 (21.1%)	

## Data Availability

The dataset, National Cancer Database, is publicly available through the American College of Surgeons https://www.facs.org/quality-programs/cancer/ncdb (accessed on 1 February 2021). Specific data used for this study are available from the authors upon reasonable request.

## Supplementary Materials

The following supporting information can be downloaded at: https://www.mdpi.com/article/10.3390/cancers15174264/s1. Figure S1a: Survival curve by HER2 expression level (IHC0, IHC1+, IHC2+) and hormonal receptor status; Figure S1b: Survival curve by HER2 expression level (IHC0, IHC1+, IHC2+) and type of treatment for HR+ breast cancer; Figure S1c: Survival curve by HER2 expression level (IHC0, IHC1+, IHC2+) and type of treatment for HR- breast cancer; Figure S2: Adjusted hazard ratio (HER2-low vs. HER2-zero breast cancer) in resectable breast cancer. (Sensitivity analysis in patients whose initial diagnosis and first-course treatment were given at the same reporting facility); Figure S3a: Adjusted hazard ratio (HER2-low vs. HER2-zero breast cancer) in HR+ resectable breast cancer. (Sensitivity analysis in patients whose initial diagnosis and first-course treatment were given at the same reporting facility); Figure S3b: Adjusted hazard ratio (HER2-low vs. HER2-zero breast cancer) in HR- resectable breast cancer. (Sensitivity analysis in patients whose initial diagnosis and first-course treatment were given at the same reporting facility); Table S1A: Characteristics of patients with HER2-low and HER2-zero HR+ resectable breast cancer; Table S1B: Characteristics of patients with HER2-low and HER2-zero HR- resectable breast cancer; Table S2: Characteristics of patients with HER2-low and HER2-zero resectable breast cancer (Sensitivity analysis in patients whose initial diagnosis and first-course treatment were given at the same reporting facility).

## References

[1] Hudis C.A. (2007). Trastuzumab—Mechanism of action and use in clinical practice. N. Engl. J. Med..

[2] Von Minckwitz G., Procter M., de Azambuja E., Zardavas D., Benyunes M., Viale G., Suter T., Arahmani A., Rouchet N., Clark E. (2017). Adjuvant Pertuzumab and Trastuzumab in Early HER2-Positive Breast Cancer. N. Engl. J. Med..

[3] Von Minckwitz G., Huang C.S., Mano M.S., Loibl S., Mamounas E.P., Untch M., Wolmark N., Rastogi P., Schneeweiss A., Redondo A. (2019). Trastuzumab Emtansine for Residual Invasive HER2-Positive Breast Cancer. N. Engl. J. Med..

[4] Wolff A.C., Hammond M.E.H., Allison K.H., Harvey B.E., Mangu P.B., Bartlett J.M., Bilous M., Ellis I.O., Fitzgibbons P., Hanna W. (2018). Human epidermal growth factor receptor 2 testing in breast cancer: American Society of Clinical Oncology/College of American Pathologists clinical practice guideline focused update. Arch. Pathol. Lab. Med..

[5] Fehrenbacher L., Cecchini R.S., Geyer C.E., Rastogi P., Costantino J.P., Atkins J.N., Crown J.P., Polikoff J., Boileau J.-F., Provencher L. (2020). NSABP B-47/NRG oncology phase III randomized trial comparing adjuvant chemotherapy with or without trastuzumab in high-risk invasive breast cancer negative for HER2 by FISH and with IHC 1+ or 2+. J. Clin. Oncol..

[6] Tarantino P., Hamilton E., Tolaney S.M., Cortes J., Morganti S., Ferraro E., Marra A., Viale G., Trapani D., Cardoso F. (2020). HER2-low breast cancer: Pathological and clinical landscape. J. Clin. Oncol..

[7] Diéras V., Deluche E., Lusque A., Pistilli B., Bachelot T., Pierga J.-Y., Viret F., Levy C., Salabert L., Du F.L. (2022). Abstract PD8-02: Trastuzumab deruxtecan (T-DXd) for advanced breast cancer patients (ABC), regardless HER2 status: A phase II study with biomarkers analysis (DAISY). Cancer Res..

[8] Modi S., Park H., Murthy R.K., Iwata H., Tamura K., Tsurutani J., Moreno-Aspitia A., Doi T., Sagara Y., Redfern C. (2020). Antitumor activity and safety of trastuzumab deruxtecan in patients with HER2-low–expressing advanced breast cancer: Results from a phase Ib study. J. Clin. Oncol..

[9] Nakada T., Sugihara K., Jikoh T., Abe Y., Agatsuma T. (2019). The latest research and development into the antibody–drug conjugate, [fam-] trastuzumab deruxtecan (DS-8201a), for HER2 cancer therapy. Chem. Pharm. Bull..

[10] Saura C., Thistlethwaite F., Banerji U., Lord S., Moreno V., MacPherson I., Boni V., Rolfo C.D., de Vries E.G., Van Herpen C.M. (2018). A phase I expansion cohorts study of SYD985 in heavily pretreated patients with HER2-positive or HER2-low metastatic breast cancer. J. Clin. Oncol..

[11] Modi S., Jacot W., Yamashita T., Sohn J., Vidal M., Tokunaga E., Tsurutani J., Ueno N.T., Prat A., Chae Y.S. (2022). Trastuzumab Deruxtecan in Previously Treated HER2-Low Advanced Breast Cancer. N. Engl. J. Med..

[12] Roy A.M., Kumarasamy V.M., Dhakal A., O’Regan R., Gandhi S. (2023). A review of treatment options in HER2-low breast cancer and proposed treatment sequencing algorithm. Cancer.

[13] Bao K.K., Sutanto L., Shirley S., Cheung K.M., Chan J.C. (2021). The association of ERBB2-low expression with the efficacy of cyclin-dependent kinase 4/6 inhibitor in hormone receptor–positive, ERBB2-negative metastatic breast cancer. JAMA Netw. Open.

[14] Dehghani M., Keshavarz P., Talei A., Akrami M., Tahmasebi S., Safaie A., Ghanbari M. (2020). The effects of low HER2/neu expression on the clinicopathological characteristics of triple-negative breast cancer patients. Asian Pac. J. Cancer Prev..

[15] Eggemann H., Ignatov T., Burger E., Kantelhardt E.J., Fettke F., Thomssen C., Costa S.D., Ignatov A. (2015). Moderate HER2 expression as a prognostic factor in hormone receptor positive breast cancer. Endocr. Relat. Cancer.

[16] Schettini F., Chic N., Brasó-Maristany F., Paré L., Pascual T., Conte B., Martínez-Sáez O., Adamo B., Vidal M., Barnadas E. (2021). Clinical, pathological, and PAM50 gene expression features of HER2-low breast cancer. npj Breast Cancer.

[17] Li Y., Abudureheiyimu N., Mo H., Guan X., Lin S., Wang Z., Chen Y., Chen S., Li Q., Cai R. (2022). In real life, low-level HER2 expression may be associated with better outcome in HER2 negative breast cancer: A study of the National Cancer Center, China. Front. Oncol..

[18] Won H.S., Ahn J., Kim Y., Kim J.S., Song J.Y., Kim H.K., Lee J., Park H.K., Kim Y.S. (2022). Clinical significance of HER2-low expression in early breast cancer: A nationwide study from the Korean Breast Cancer Society. Breast Cancer Res..

[19] Shang J., Sun X., Xu Z., Cai L., Liu C., Wu S., Liu Y. (2023). Evolution and clinical significance of HER2-low status after neoadjuvant therapy for breast cancer. Front. Oncol..

[20] Miglietta F., Griguolo G., Bottosso M., Giarratano T., Lo Mele M., Fassan M., Cacciatore M., Genovesi E., De Bartolo D., Vernaci G. (2022). HER2-low-positive breast cancer: Evolution from primary tumor to residual disease after neoadjuvant treatment. npj Breast Cancer.

[21] Tarantino P., Jin Q., Tayob N., Jeselsohn R.M., Schnitt S.J., Vincuilla J., Parker T., Tyekucheva S., Li T., Lin N.U. (2022). Prognostic and Biologic Significance of ERBB2-Low Expression in Early-Stage Breast Cancer. JAMA Oncol..

[22] Sparano J.A., Gray R.J., Makower D.F., Pritchard K.I., Albain K.S., Hayes D.F., Geyer C.E., Dees E.C., Goetz M.P., Olson J.A. (2018). Adjuvant Chemotherapy Guided by a 21-Gene Expression Assay in Breast Cancer. N. Engl. J. Med..

[23] Puglisi F., Gerratana L., Lambertini M., Ceppi M., Boni L., Montemurro F., Russo S., Bighin C., De Laurentiis M., Giuliano M. (2021). Composite risk and benefit from adjuvant dose-dense chemotherapy in hormone receptor-positive breast cancer. npj Breast Cancer.

[24] Licata L., Viale G., Giuliano M., Curigliano G., Chavez-MacGregor M., Foldi J., Oke O., Collins J., Del Mastro L., Puglisi F. (2023). Oncotype DX results increase concordance in adjuvant chemotherapy recommendations for early-stage breast cancer. npj Breast Cancer.

[25] Garutti M., Griguolo G., Botticelli A., Buzzatti G., De Angelis C., Gerratana L., Molinelli C., Adamo V., Bianchini G., Biganzoli L. (2022). Definition of High-Risk Early Hormone-Positive HER2-Negative Breast Cancer: A Consensus Review. Cancers.

[26] Peiffer D.S., Zhao F., Chen N., Hahn O.M., Nanda R., Olopade O.I., Huo D., Howard F.M. (2023). Clinicopathologic Characteristics and Prognosis of ERBB2-Low Breast Cancer Among Patients in the National Cancer Database. JAMA Oncol..

[27] Rusthoven C.G., Rabinovitch R.A., Jones B.L., Koshy M., Amini A., Yeh N., Jackson M.W., Fisher C.M. (2016). The impact of postmastectomy and regional nodal radiation after neoadjuvant chemotherapy for clinically lymph node-positive breast cancer: A National Cancer Database (NCDB) analysis. Ann. Oncol..

[28] Zeidman M., Alberty-Oller J.J., Ru M., Pisapati K.V., Moshier E., Ahn S., Mazumdar M., Port E., Schmidt H. (2020). Use of neoadjuvant versus adjuvant chemotherapy for hormone receptor-positive breast cancer: A National Cancer Database (NCDB) study. Breast Cancer Res. Treat..

[29] Fayanju O.M., Ren Y., Thomas S.M., Greenup R.A., Plichta J.K., Rosenberger L.H., Tamirisa N., Force J., Boughey J.C., Hyslop T. (2018). The Clinical Significance of Breast-only and Node-only Pathologic Complete Response (pCR) After Neoadjuvant Chemotherapy (NACT): A Review of 20,000 Breast Cancer Patients in the National Cancer Data Base (NCDB). Ann. Surg..

[30] Von Minckwitz G., Untch M., Blohmer J.U., Costa S.D., Eidtmann H., Fasching P.A., Gerber B., Eiermann W., Hilfrich J., Huober J. (2012). Definition and impact of pathologic complete response on prognosis after neoadjuvant chemotherapy in various intrinsic breast cancer subtypes. J. Clin. Oncol..

[31] Denkert C., Seither F., Schneeweiss A., Link T., Blohmer J.U., Just M., Wimberger P., Forberger A., Tesch H., Jackisch C. (2021). Clinical and molecular characteristics of HER2-low-positive breast cancer: Pooled analysis of individual patient data from four prospective, neoadjuvant clinical trials. Lancet Oncol..

[32] Zhang H., Katerji H., Turner B.M., Audeh W., Hicks D.G. (2022). HER2-low breast cancers: Incidence, HER2 staining patterns, clinicopathologic features, MammaPrint and BluePrint genomic profiles. Mod. Pathol..

[33] Catalfamo K., Attwood K., Kapoor A., Jatwani K., Roy A.M. (2023). Racial disparity in the clinical outcomes of HER2-low and HER2-zero early-stage breast cancer. J. Clin. Oncol..

[34] Chang J., Ormerod M., Powles T., Allred D., Ashley S., Dowsett M. (2000). Apoptosis and proliferation as predictors of chemotherapy response in patients with breast carcinoma. Cancer Interdiscip. Int. J. Am. Cancer Soc..

[35] Amadori D., Volpi A., Maltoni R., Nanni O., Amaducci L., Amadori A., Giunchi D.C., Vio A., Saragoni A., Silvestrini R. (1997). Cell proliferation as a predictor of response to chemotherapy in metastatic breast cancer: A prospective study. Breast Cancer Res. Treat..

[36] Mutai R., Barkan T., Moore A., Sarfaty M., Shochat T., Yerushalmi R., Stemmer S.M., Goldvaser H. (2021). Prognostic impact of HER2-low expression in hormone receptor positive early breast cancer. Breast.

[37] Jiang C., Perimbeti S., Deng L., Shapiro C.L., Gandhi S. (2022). Clinical outcomes of de novo metastatic HER2-low breast cancer: A National Cancer Database Analysis. npj Breast Cancer.

[38] American College of Surgeons National Cancer Database. https://www.facs.org/quality-programs/cancer/ncdb.

[39] American College of Surgery PUF Data Dictionary. https://www.facs.org/quality-programs/cancer/ncdb/puf.

[40] Fernandez A.I., Liu M., Bellizzi A., Brock J., Fadare O., Hanley K., Harigopal M., Jorns J.M., Kuba M.G., Ly A. (2022). Examination of Low ERBB2 Protein Expression in Breast Cancer Tissue. JAMA Oncol..

[41] Wolff A.C., Hammond M.E., Hicks D.G., Dowsett M., McShane L.M., Allison K.H., Allred D.C., Bartlett J.M., Bilous M., Fitzgibbons P. (2013). Recommendations for human epidermal growth factor receptor 2 testing in breast cancer: American Society of Clinical Oncology/College of American Pathologists clinical practice guideline update. J. Clin. Oncol..

[42] Gupta R.K., Roy A.M., Gupta A., Takabe K., Dhakal A., Opyrchal M., Kalinski P., Gandhi S. (2022). Systemic Therapy De-Escalation in Early-Stage Triple-Negative Breast Cancer: Dawn of a New Era?. Cancers.

[43] Akrida I., Mulita F. (2022). The clinical significance of HER2 expression in DCIS. Med. Oncol..

