# Supplementary material for: Oncotype Dx Score, HER2 Low Expression, and Clinical Outcomes in Early-Stage Breast Cancer: A National Cancer Database Analysis
Source: Cancers (Basel). 2023 Aug 25;15(17):4264. doi: 10.3390/cancers15174264 (PMC10486548; doi:10.3390/cancers15174264)
Supplement: Supplementary file 1 [file cancers-15-04264-s001.zip › Supplementary figure.pptx]

## Slide 1
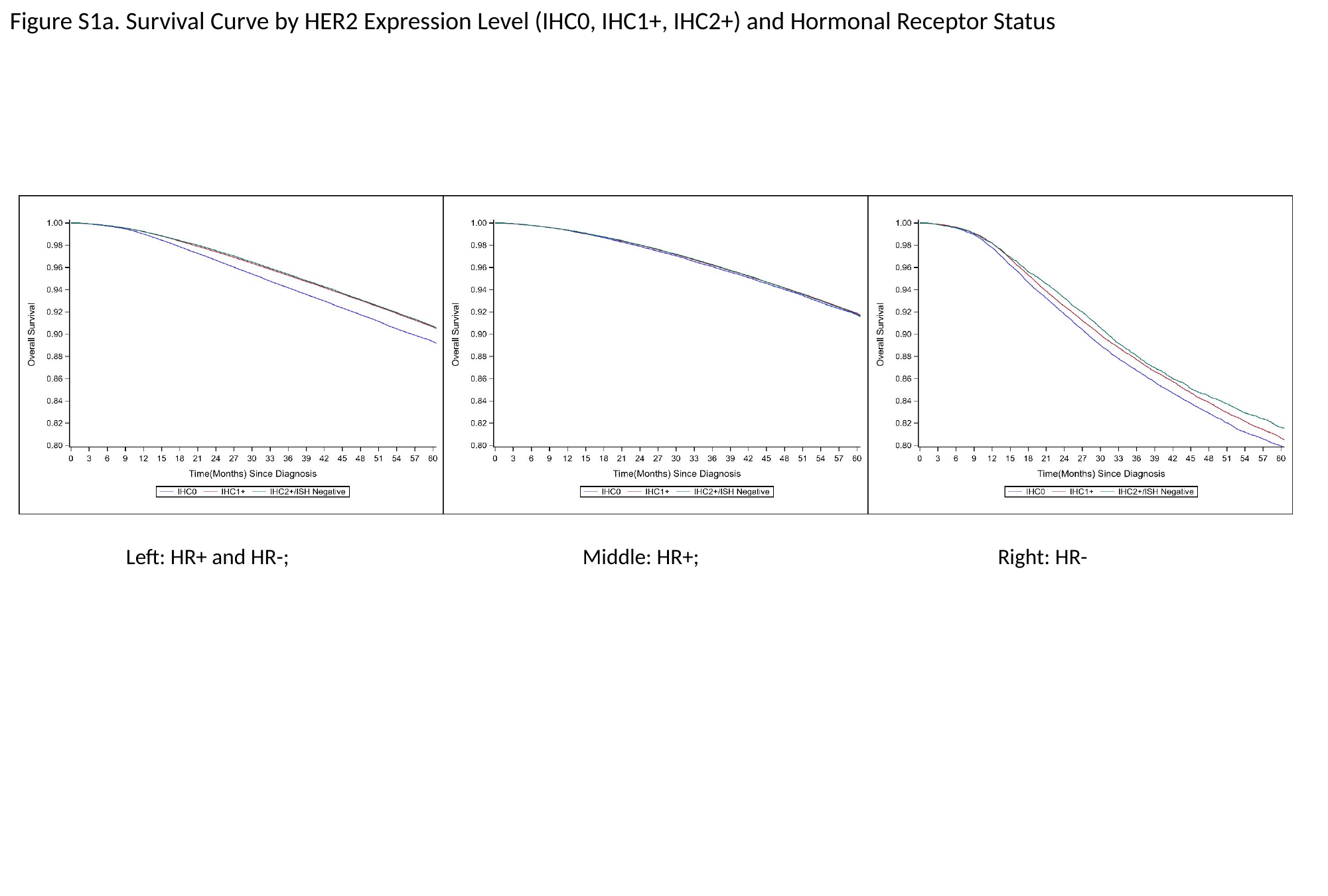

Figure S1a. Survival Curve by HER2 Expression Level (IHC0, IHC1+, IHC2+) and Hormonal Receptor Status
Left: HR+ and HR-; Middle: HR+; Right: HR-

## Slide 2
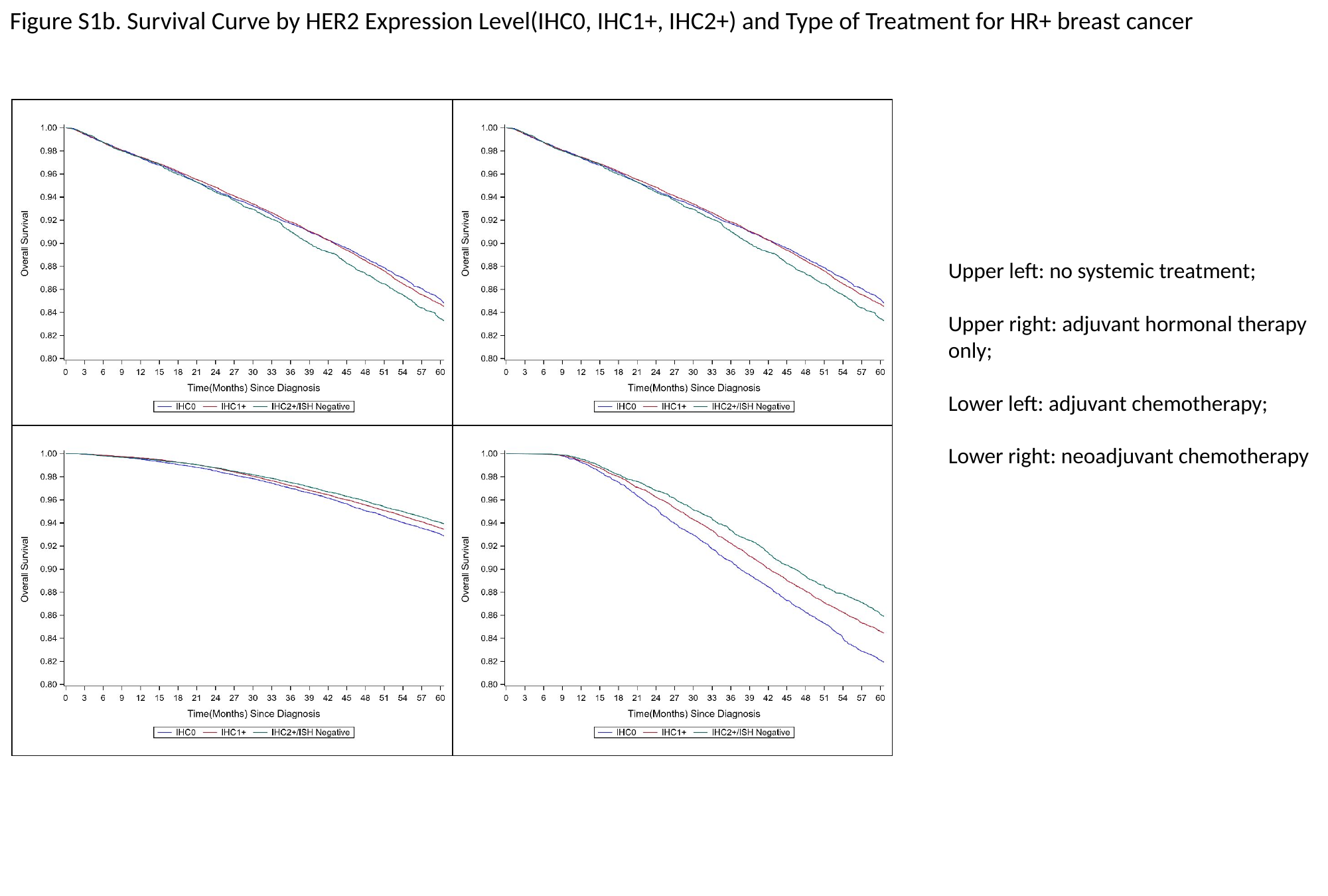

Figure S1b. Survival Curve by HER2 Expression Level(IHC0, IHC1+, IHC2+) and Type of Treatment for HR+ breast cancer
Upper left: no systemic treatment;
Upper right: adjuvant hormonal therapy only;
Lower left: adjuvant chemotherapy;
Lower right: neoadjuvant chemotherapy

## Slide 3
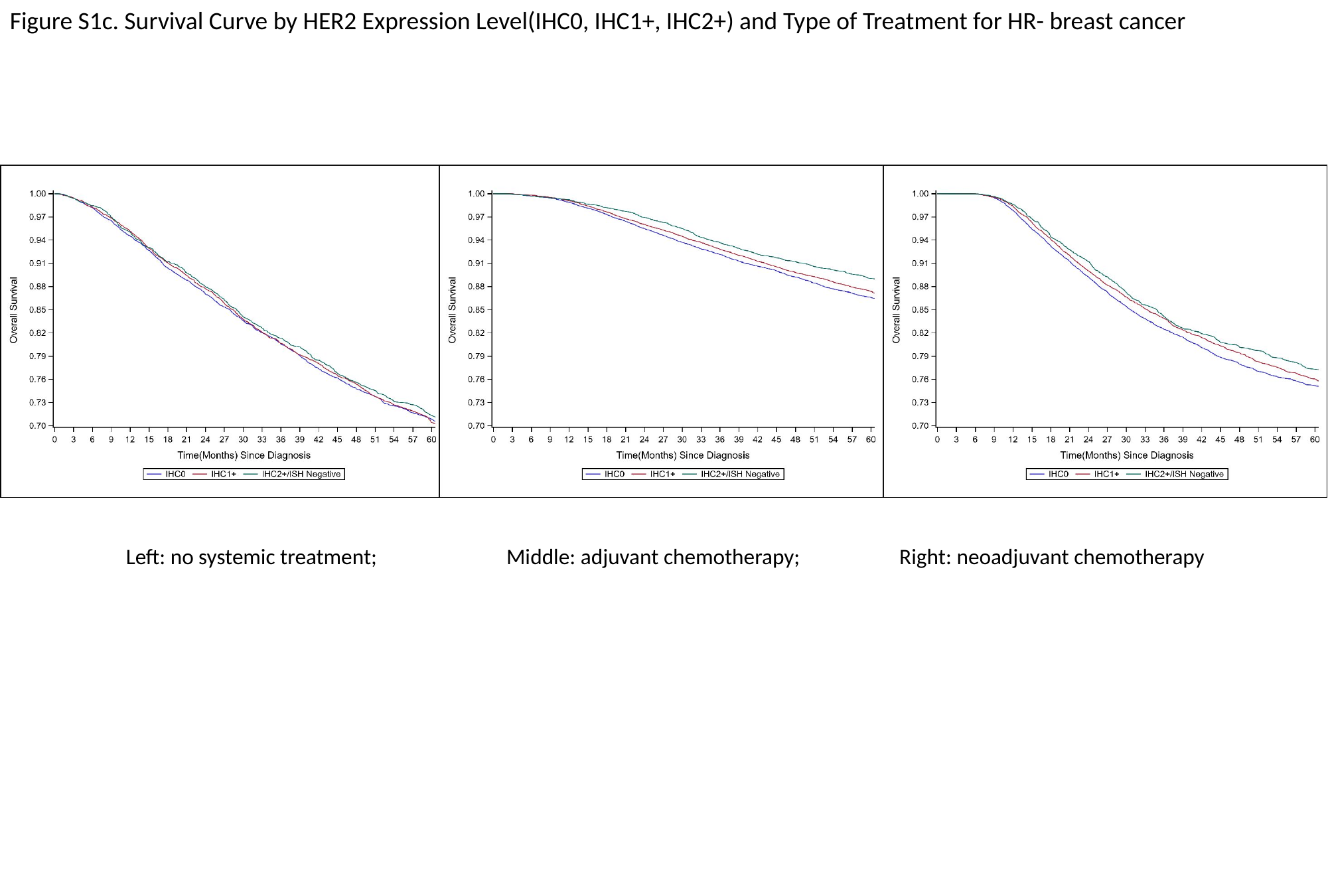

Figure S1c. Survival Curve by HER2 Expression Level(IHC0, IHC1+, IHC2+) and Type of Treatment for HR- breast cancer
Left: no systemic treatment; Middle: adjuvant chemotherapy; Right: neoadjuvant chemotherapy

## Slide 4
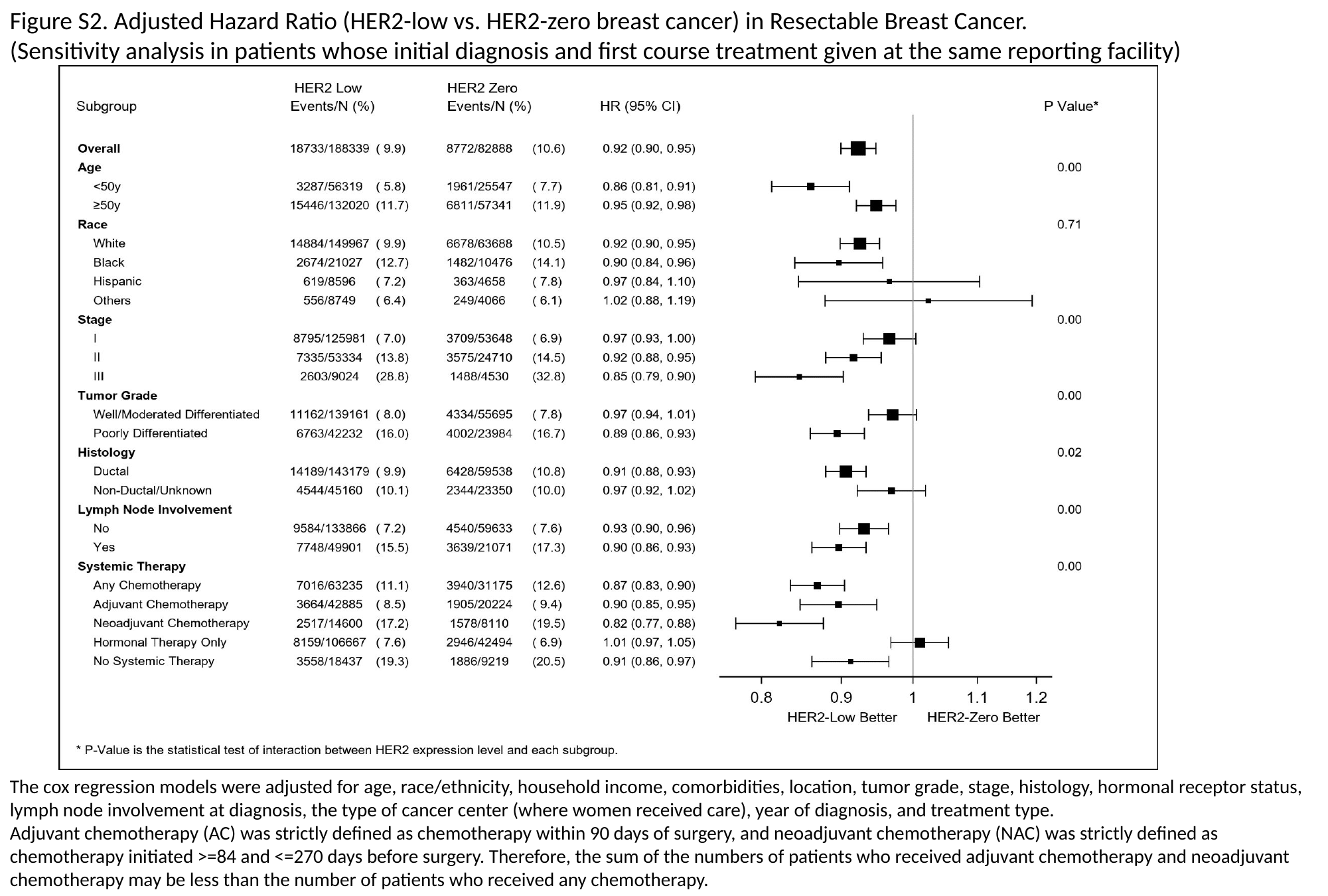

Figure S2. Adjusted Hazard Ratio (HER2-low vs. HER2-zero breast cancer) in Resectable Breast Cancer.
(Sensitivity analysis in patients whose initial diagnosis and first course treatment given at the same reporting facility)
The cox regression models were adjusted for age, race/ethnicity, household income, comorbidities, location, tumor grade, stage, histology, hormonal receptor status, lymph node involvement at diagnosis, the type of cancer center (where women received care), year of diagnosis, and treatment type.
Adjuvant chemotherapy (AC) was strictly defined as chemotherapy within 90 days of surgery, and neoadjuvant chemotherapy (NAC) was strictly defined as chemotherapy initiated >=84 and <=270 days before surgery. Therefore, the sum of the numbers of patients who received adjuvant chemotherapy and neoadjuvant chemotherapy may be less than the number of patients who received any chemotherapy.

## Slide 5
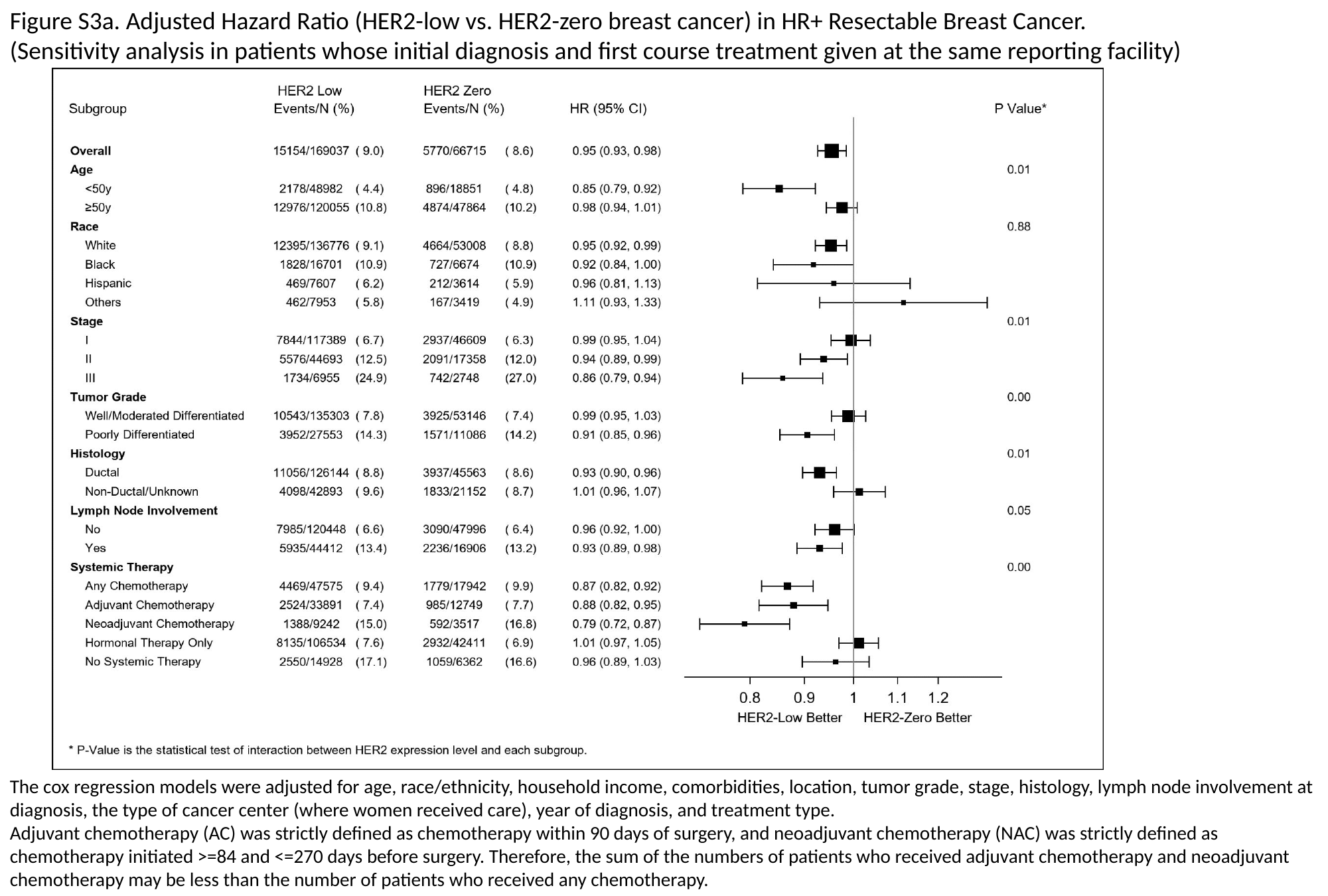

Figure S3a. Adjusted Hazard Ratio (HER2-low vs. HER2-zero breast cancer) in HR+ Resectable Breast Cancer.
(Sensitivity analysis in patients whose initial diagnosis and first course treatment given at the same reporting facility)
The cox regression models were adjusted for age, race/ethnicity, household income, comorbidities, location, tumor grade, stage, histology, lymph node involvement at diagnosis, the type of cancer center (where women received care), year of diagnosis, and treatment type.
Adjuvant chemotherapy (AC) was strictly defined as chemotherapy within 90 days of surgery, and neoadjuvant chemotherapy (NAC) was strictly defined as chemotherapy initiated >=84 and <=270 days before surgery. Therefore, the sum of the numbers of patients who received adjuvant chemotherapy and neoadjuvant chemotherapy may be less than the number of patients who received any chemotherapy.

## Slide 6
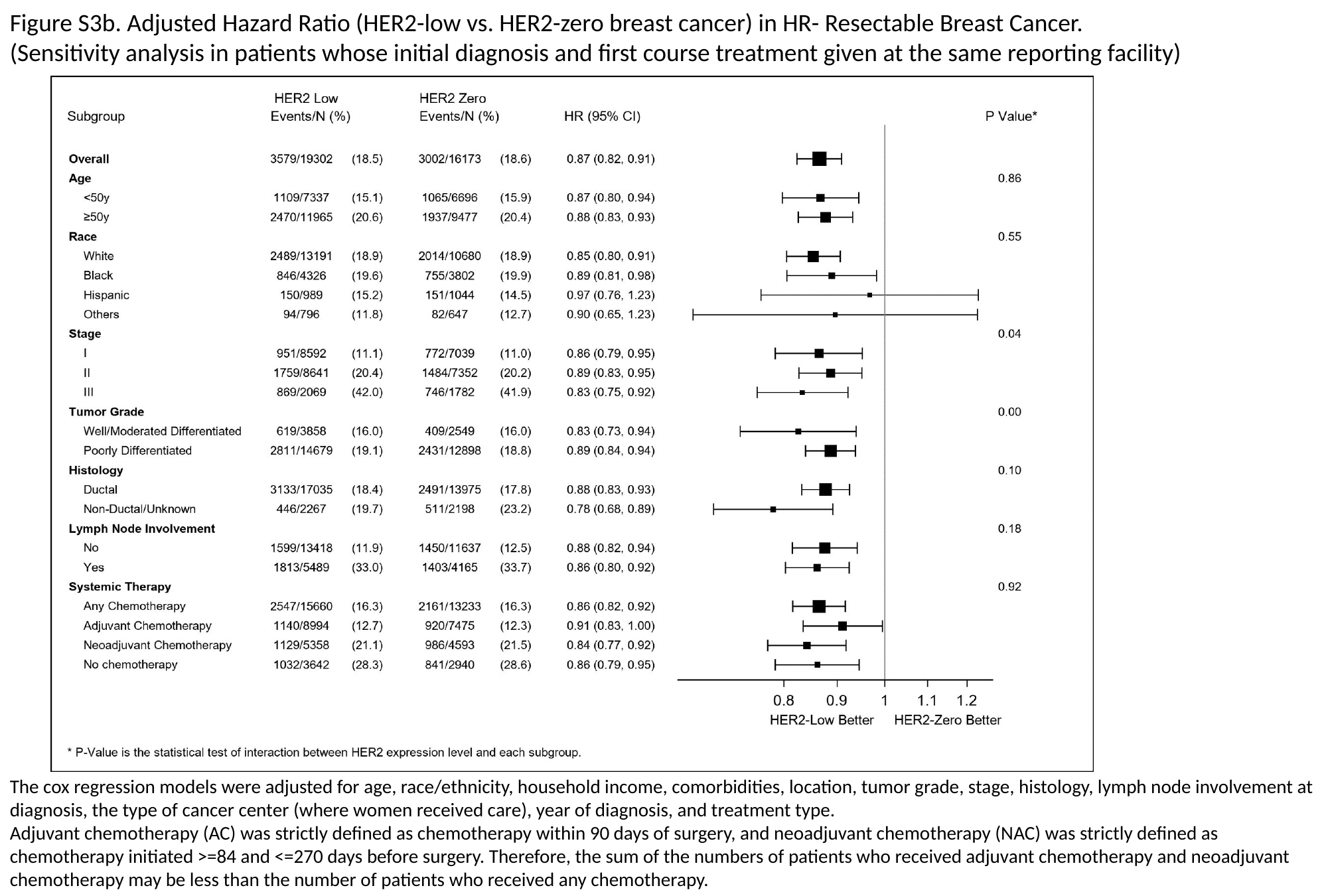

Figure S3b. Adjusted Hazard Ratio (HER2-low vs. HER2-zero breast cancer) in HR- Resectable Breast Cancer.
(Sensitivity analysis in patients whose initial diagnosis and first course treatment given at the same reporting facility)
The cox regression models were adjusted for age, race/ethnicity, household income, comorbidities, location, tumor grade, stage, histology, lymph node involvement at diagnosis, the type of cancer center (where women received care), year of diagnosis, and treatment type.
Adjuvant chemotherapy (AC) was strictly defined as chemotherapy within 90 days of surgery, and neoadjuvant chemotherapy (NAC) was strictly defined as chemotherapy initiated >=84 and <=270 days before surgery. Therefore, the sum of the numbers of patients who received adjuvant chemotherapy and neoadjuvant chemotherapy may be less than the number of patients who received any chemotherapy.
