# Supplementary material for: Oncotype Dx Score, HER2 Low Expression, and Clinical Outcomes in Early-Stage Breast Cancer: A National Cancer Database Analysis
Source: Cancers (Basel). 2023 Aug 25;15(17):4264. doi: 10.3390/cancers15174264 (PMC10486548; doi:10.3390/cancers15174264)
Supplement: Supplementary file 1 [file cancers-15-04264-s001.zip › Supplementary TableS1B.pdf]

TableS1B. Characteristics of Patients with HER2-Low and HER2-Zero HR- Resectable Breast Cancer.

Variable	Level	N	Overall N=75822	Her2 Low N=40052	Her2 Zero N=35770	P-Value
Age		75822	57.5 ± 13.5	58.1 ± 13.5	56.8 ± 13.5	**<.001**
Race	1. White	75822	51116 (67.4%)	27275 (68.1%)	23841 (66.7%)	**<.001**
	2. Black		16487 (21.7%)	8630 (21.5%)	7857 (22.0%)	
	3. Hispanic		4806 (6.3%)	2285 (5.7%)	2521 (7.0%)	
	4. Asian and Pacific Islanders		2371 (3.1%)	1314 (3.3%)	1057 (3.0%)	
	5. Other or unknown		1042 (1.4%)	548 (1.4%)	494 (1.4%)	
Insurance	1. Private	75822	42332 (55.8%)	21871 (54.6%)	20461 (57.2%)	**<.001**
	2. Public Insurance		30884 (40.7%)	16853 (42.1%)	14031 (39.2%)	
	3. Uninsured		1839 (2.4%)	934 (2.3%)	905 (2.5%)	
	4. Unknown		767 (1.0%)	394 (1.0%)	373 (1.0%)	
Household Income	1. <$40,227	75822	12742 (16.8%)	6677 (16.7%)	6065 (17.0%)	**<.001**
	2. $40,227 - $50,353		13733 (18.1%)	7369 (18.4%)	6364 (17.8%)	
	3. $50,354 - $63,332		15102 (19.9%)	7874 (19.7%)	7228 (20.2%)	
	4. >=$63,333		24177 (31.9%)	12486 (31.2%)	11691 (32.7%)	
	5. Unknown		10068 (13.3%)	5646 (14.1%)	4422 (12.4%)	
Treatment Setting	1. Community Cancer Program	75822	4500 (5.9%)	2471 (6.2%)	2029 (5.7%)	**<.001**
	2. Comprehensive Community Cancer Program		27475 (36.2%)	15170 (37.9%)	12305 (34.4%)	
	3. Academic Comprehensive Cancer Program		21950 (28.9%)	10938 (27.3%)	11012 (30.8%)	
	4. Integrated Network Cancer Program		14649 (19.3%)	7971 (19.9%)	6678 (18.7%)	
	5. Unknown		7248 (9.6%)	3502 (8.7%)	3746 (10.5%)	
Treatment Location	1. Metro	75822	64073 (84.5%)	33648 (84.0%)	30425 (85.1%)	**<.001**
	2. Urban		8738 (11.5%)	4787 (12.0%)	3951 (11.0%)	
	3. Rural		1133 (1.5%)	645 (1.6%)	488 (1.4%)	
	4. Unknown		1878 (2.5%)	972 (2.4%)	906 (2.5%)	

TableS1B. Characteristics of Patients with HER2-Low and HER2-Zero HR- Resectable Breast Cancer.

**Variable**	**Level**	**N**	**Overall N=75822**	**Her2 Low N=40052**	**Her2 Zero N=35770**	**P-Value**
Histology	1. Ductal adenocarcinoma	75822	66564 (87.8%)	35418 (88.4%)	31146 (87.1%)	**<.001**
	2. Lobular adenocarcinoma		699 (0.9%)	462 (1.2%)	237 (0.7%)	
	3. Mixed or unknown histology		8559 (11.3%)	4172 (10.4%)	4387 (12.3%)	
Tumor Grade	1. Well differentiated	75822	1281 (1.7%)	719 (1.8%)	562 (1.6%)	**<.001**
	2. Moderately differentiated		11667 (15.4%)	6880 (17.2%)	4787 (13.4%)	
	3. Poorly differentiated/Undifferentiated		59796 (78.9%)	30870 (77.1%)	28926 (80.9%)	
	4. Unknown		3078 (4.1%)	1583 (4.0%)	1495 (4.2%)	
Clinical Stage	Stage I	75822	32421 (42.8%)	17254 (43.1%)	15167 (42.4%)	0.051
	Stage II		34725 (45.8%)	18176 (45.4%)	16549 (46.3%)	
	Stage III		8676 (11.4%)	4622 (11.5%)	4054 (11.3%)	
Lymph Node Involvement	1. No lymph node	75822	53054 (70.0%)	27507 (68.7%)	25547 (71.4%)	**<.001**
	2. 1-3 lymph nodes		15018 (19.8%)	8245 (20.6%)	6773 (18.9%)	
	3. 4+ lymph nodes		6281 (8.3%)	3536 (8.8%)	2745 (7.7%)	
	4. Unknown		1469 (1.9%)	764 (1.9%)	705 (2.0%)	
Hormonal Receptor Status	2. No	75822	75822 (100.0%)	40052 (100.0%)	35770 (100.0%)	-
Surgical Treatment	1. Lumpectomy or partial mastectomy	75822	41383 (54.6%)	21715 (54.2%)	19668 (55.0%)	**0.034**
	2. Total mastectomy		34439 (45.4%)	18337 (45.8%)	16102 (45.0%)	
Adjuvant Radiation	1. Yes	75822	49457 (65.2%)	26193 (65.4%)	23264 (65.0%)	0.299
	2. No		26365 (34.8%)	13859 (34.6%)	12506 (35.0%)	
Chemotherapy	1. Yes	75822	63251 (83.4%)	33253 (83.0%)	29998 (83.9%)	**0.002**
	2. No		12571 (16.6%)	6799 (17.0%)	5772 (16.1%)	
Neoadjuvant Chemotherapy	1. Yes	75822	22621 (29.8%)	11847 (29.6%)	10774 (30.1%)	0.104
	2. No		53201 (70.2%)	28205 (70.4%)	24996 (69.9%)	

TableS1B. Characteristics of Patients with HER2-Low and HER2-Zero HR- Resectable Breast Cancer.

**Variable**	**Level**	**N**	**Overall N=75822**	**Her2 Low N=40052**	**Her2 Zero N=35770**	**P-Value**
Adjuvant Chemotherapy	1. Yes	75822	34917 (46.1%)	18477 (46.1%)	16440 (46.0%)	0.635
	2. No		40905 (53.9%)	21575 (53.9%)	19330 (54.0%)	
Comorbidity Score	0	75822	62613 (82.6%)	33064 (82.6%)	29549 (82.6%)	0.618
	1		10123 (13.4%)	5364 (13.4%)	4759 (13.3%)	
	2		2143 (2.8%)	1144 (2.9%)	999 (2.8%)	
	>=3		943 (1.2%)	480 (1.2%)	463 (1.3%)	
Year of Diagnosis	2010	75822	7092 (9.4%)	4033 (10.1%)	3059 (8.6%)	**<.001**
	2011		8112 (10.7%)	4598 (11.5%)	3514 (9.8%)	
	2012		8630 (11.4%)	4917 (12.3%)	3713 (10.4%)	
	2013		9419 (12.4%)	5053 (12.6%)	4366 (12.2%)	
	2014		9877 (13.0%)	5299 (13.2%)	4578 (12.8%)	
	2015		10679 (14.1%)	5606 (14.0%)	5073 (14.2%)	
	2016		10953 (14.4%)	5437 (13.6%)	5516 (15.4%)	
	2017		11060 (14.6%)	5109 (12.8%)	5951 (16.6%)	
