# Supplementary material for: Oncotype Dx Score, HER2 Low Expression, and Clinical Outcomes in Early-Stage Breast Cancer: A National Cancer Database Analysis
Source: Cancers (Basel). 2023 Aug 25;15(17):4264. doi: 10.3390/cancers15174264 (PMC10486548; doi:10.3390/cancers15174264)
Supplement: Supplementary file 1 [file cancers-15-04264-s001.zip › Supplementary TableS2.pdf]

TableS2. Characteristics of Patients with HER2-Low and HER2-Zero Resectable Breast Cancer.
(Sensitivity analysis in patients whose initial diagnosis and first course treatment given at the same reporting facility).

Variable	Level	N	Overall N=271227	Her2 Low N=188339	Her2 Zero N=82888	P-Value
Age		271227	61.4 ±12.6	61.5 ±12.5	61.2 ±12.7	-
Race	1. White	271227	213655 (78.8%)	149967 (79.6%)	63688 (76.8%)	**<.001**
	2. Black		31503 (11.6%)	21027 (11.2%)	10476 (12.6%)	
	3. Hispanic		13254 (4.9%)	8596 (4.6%)	4658 (5.6%)	
	4. Asian and Pacific Islanders		9692 (3.6%)	6684 (3.5%)	3008 (3.6%)	
	5. Other or unknown		3123 (1.2%)	2065 (1.1%)	1058 (1.3%)	
Insurance	1. Private	271227	138386 (51.0%)	95740 (50.8%)	42646 (51.5%)	**<.001**
	2. Public Insurance		125753 (46.4%)	87882 (46.7%)	37871 (45.7%)	
	3. Uninsured		4935 (1.8%)	3283 (1.7%)	1652 (2.0%)	
	4. Unknown		2153 (0.8%)	1434 (0.8%)	719 (0.9%)	
Household Income	1. <$40,227	271227	36394 (13.4%)	24927 (13.2%)	11467 (13.8%)	**<.001**
	2. $40,227 - $50,353		47411 (17.5%)	33008 (17.5%)	14403 (17.4%)	
	3. $50,354 - $63,332		55339 (20.4%)	38457 (20.4%)	16882 (20.4%)	
	4. >=$63,333		94402 (34.8%)	64408 (34.2%)	29994 (36.2%)	
	5. Unknown		37681 (13.9%)	27539 (14.6%)	10142 (12.2%)	
Treatment Setting	1. Community Cancer Program	271227	21278 (7.8%)	15280 (8.1%)	5998 (7.2%)	**<.001**
	2. Comprehensive Community Cancer Program		115039 (42.4%)	82021 (43.5%)	33018 (39.8%)	
	3. Academic Comprehensive Cancer Program		65764 (24.2%)	42377 (22.5%)	23387 (28.2%)	
	4. Integrated Network Cancer Program		58428 (21.5%)	41585 (22.1%)	16843 (20.3%)	
	5. Unknown		10718 (4.0%)	7076 (3.8%)	3642 (4.4%)	
Treatment Location	1. Metro	271227	234368 (86.4%)	162045 (86.0%)	72323 (87.3%)	**<.001**
	2. Urban		29658 (10.9%)	21192 (11.3%)	8466 (10.2%)	
	3. Rural		3600 (1.3%)	2653 (1.4%)	947 (1.1%)	
	4. Unknown		3601 (1.3%)	2449 (1.3%)	1152 (1.4%)	

TableS2. Characteristics of Patients with HER2-Low and HER2-Zero Resectable Breast Cancer. (Sensitivity analysis in patients whose initial diagnosis and first course treatment given at the same reporting facility).

Variable	Level	N	Overall N=271227	Her2 Low N=188339	Her2 Zero N=82888	P-Value
Histology	1. Ductal adenocarcinoma	271227	202717 (74.7%)	143179 (76.0%)	59538 (71.8%)	**<.001**
	2. Lobular adenocarcinoma		28700 (10.6%)	19190 (10.2%)	9510 (11.5%)	
	3. Mixed or unknown histology		39810 (14.7%)	25970 (13.8%)	13840 (16.7%)	
Tumor Grade	1. Well differentiated	271227	75179 (27.7%)	53806 (28.6%)	21373 (25.8%)	**<.001**
	2. Moderately differentiated		119677 (44.1%)	85355 (45.3%)	34322 (41.4%)	
	3. Poorly differentiated/Undifferentiated		66216 (24.4%)	42232 (22.4%)	23984 (28.9%)	
	4. Unknown		10155 (3.7%)	6946 (3.7%)	3209 (3.9%)	
Clinical Stage	Stage I	271227	179629 (66.2%)	125981 (66.9%)	53648 (64.7%)	**<.001**
	Stage II		78044 (28.8%)	53334 (28.3%)	24710 (29.8%)	
	Stage III		13554 (5.0%)	9024 (4.8%)	4530 (5.5%)	
Lymph Node Involvement	1. No lymph node	271227	193499 (71.3%)	133866 (71.1%)	59633 (71.9%)	**<.001**
	2. 1-3 lymph nodes		53302 (19.7%)	37528 (19.9%)	15774 (19.0%)	
	3. 4+ lymph nodes		17670 (6.5%)	12373 (6.6%)	5297 (6.4%)	
	4. Unknown		6756 (2.5%)	4572 (2.4%)	2184 (2.6%)	
Hormonal Receptor Status	1. Yes	271227	235752 (86.9%)	169037 (89.8%)	66715 (80.5%)	**<.001**
	2. No		35475 (13.1%)	19302 (10.2%)	16173 (19.5%)	
Surgical Treatment	1. Lumpectomy or partial mastectomy	271227	176483 (65.1%)	122191 (64.9%)	54292 (65.5%)	**0.002**
	2. Total mastectomy		94744 (34.9%)	66148 (35.1%)	28596 (34.5%)	
Adjuvant Radiation	1. Yes	271227	181348 (66.9%)	125948 (66.9%)	55400 (66.8%)	0.855
	2. No		89879 (33.1%)	62391 (33.1%)	27488 (33.2%)	
Chemotherapy	1. Yes	271227	94410 (34.8%)	63235 (33.6%)	31175 (37.6%)	**<.001**
	2. No		176817 (65.2%)	125104 (66.4%)	51713 (62.4%)	
Neoadjuvant Chemotherapy	1. Yes	271227	22710 (8.4%)	14600 (7.8%)	8110 (9.8%)	**<.001**
	2. No		248517 (91.6%)	173739 (92.2%)	74778 (90.2%)	

TableS2. Characteristics of Patients with HER2-Low and HER2-Zero Resectable Breast Cancer.
(Sensitivity analysis in patients whose initial diagnosis and first course treatment given at the same reporting facility).

Variable	Level	N	Overall N=271227	Her2 Low N=188339	Her2 Zero N=82888	P-Value
Adjuvant Chemotherapy	1. Yes	271227	63109 (23.3%)	42885 (22.8%)	20224 (24.4%)	**<.001**
	2. No		208118 (76.7%)	145454 (77.2%)	62664 (75.6%)	
Hormone Treatment	1. Yes	271227	211244 (77.9%)	151794 (80.6%)	59450 (71.7%)	**<.001**
	2. No		59983 (22.1%)	36545 (19.4%)	23438 (28.3%)	
Comorbidity Score	0	271227	221728 (81.7%)	153908 (81.7%)	67820 (81.8%)	0.224
	1		37845 (14.0%)	26402 (14.0%)	11443 (13.8%)	
	2		8250 (3.0%)	5704 (3.0%)	2546 (3.1%)	
	>=3		3404 (1.3%)	2325 (1.2%)	1079 (1.3%)	
Year of Diagnosis	2010	271227	21351 (7.9%)	15164 (8.1%)	6187 (7.5%)	**<.001**
	2011		26439 (9.7%)	18960 (10.1%)	7479 (9.0%)	
	2012		29105 (10.7%)	21602 (11.5%)	7503 (9.1%)	
	2013		33208 (12.2%)	23298 (12.4%)	9910 (12.0%)	
	2014		36012 (13.3%)	25466 (13.5%)	10546 (12.7%)	
	2015		38779 (14.3%)	26789 (14.2%)	11990 (14.5%)	
	2016		42465 (15.7%)	28628 (15.2%)	13837 (16.7%)	
	2017		43868 (16.2%)	28432 (15.1%)	15436 (18.6%)	
