# Supplementary material for: Oncotype Dx Score, HER2 Low Expression, and Clinical Outcomes in Early-Stage Breast Cancer: A National Cancer Database Analysis
Source: Cancers (Basel). 2023 Aug 25;15(17):4264. doi: 10.3390/cancers15174264 (PMC10486548; doi:10.3390/cancers15174264)
Supplement: Supplementary file 1 [file cancers-15-04264-s001.zip › SupplementaryTableS1A.pdf]

TableS1A. Characteristics of Patients with HER2-Low and HER2-Zero HR+ Resectable Breast Cancer.

**Variable**	**Level**	**N**	**Overall N=477675**	**Her2 Low N=336147**	**Her2 Zero N=141528**	**P-Value**
Age		477675	61.1 ± 12.4	61.0 ± 12.4	61.3 ± 12.4	**<.001**
Race	1. White	477675	382722 (80.1%)	270123 (80.4%)	112599 (79.6%)	**<.001**
	2. Black		44442 (9.3%)	31467 (9.4%)	12975 (9.2%)	
	3. Hispanic		25186 (5.3%)	16810 (5.0%)	8376 (5.9%)	
	4. Asian and Pacific Islanders		18184 (3.8%)	12809 (3.8%)	5375 (3.8%)	
	5. Other or unknown		7141 (1.5%)	4938 (1.5%)	2203 (1.6%)	
Insurance	1. Private	477675	252011 (52.8%)	177379 (52.8%)	74632 (52.7%)	0.055
	2. Public Insurance		214193 (44.8%)	150818 (44.9%)	63375 (44.8%)	
	3. Uninsured		7113 (1.5%)	4955 (1.5%)	2158 (1.5%)	
	4. Unknown		4358 (0.9%)	2995 (0.9%)	1363 (1.0%)	
Household Income	1. <$40,227	477675	58153 (12.2%)	41065 (12.2%)	17088 (12.1%)	**<.001**
	2. $40,227 - $50,353		80854 (16.9%)	57545 (17.1%)	23309 (16.5%)	
	3. $50,354 - $63,332		96249 (20.1%)	67982 (20.2%)	28267 (20.0%)	
	4. >=$63,333		176699 (37.0%)	121483 (36.1%)	55216 (39.0%)	
	5. Unknown		65720 (13.8%)	48072 (14.3%)	17648 (12.5%)	
Treatment Setting	1. Community Cancer Program	477675	31808 (6.7%)	22931 (6.8%)	8877 (6.3%)	**<.001**
	2. Comprehensive Community Cancer Program		191996 (40.2%)	138641 (41.2%)	53355 (37.7%)	
	3. Academic Comprehensive Cancer Program		137962 (28.9%)	91607 (27.3%)	46355 (32.8%)	
	4. Integrated Network Cancer Program		97637 (20.4%)	69928 (20.8%)	27709 (19.6%)	
	5. Unknown		18272 (3.8%)	13040 (3.9%)	5232 (3.7%)	
Treatment Location	1. Metro	477675	405987 (85.0%)	284150 (84.5%)	121837 (86.1%)	**<.001**
	2. Urban		53722 (11.2%)	39216 (11.7%)	14506 (10.2%)	
	3. Rural		6922 (1.4%)	5181 (1.5%)	1741 (1.2%)	
	4. Unknown		11044 (2.3%)	7600 (2.3%)	3444 (2.4%)	

TableS1A. Characteristics of Patients with HER2-Low and HER2-Zero HR+ Resectable Breast Cancer.

Variable	Level	N	Overall N=477675	Her2 Low N=336147	Her2 Zero N=141528	P-Value
Histology	1. Ductal adenocarcinoma	477675	348183 (72.9%)	250891 (74.6%)	97292 (68.7%)	**<.001**
	2. Lobular adenocarcinoma		57764 (12.1%)	37693 (11.2%)	20071 (14.2%)	
	3. Mixed or unknown histology		71728 (15.0%)	47563 (14.1%)	24165 (17.1%)	
Tumor Grade	1. Well differentiated	477675	140809 (29.5%)	99098 (29.5%)	41711 (29.5%)	0.169
	2. Moderately differentiated		236545 (49.5%)	166697 (49.6%)	69848 (49.4%)	
	3. Poorly differentiated/Undifferentiated		83009 (17.4%)	58267 (17.3%)	24742 (17.5%)	
	4. Unknown		17312 (3.6%)	12085 (3.6%)	5227 (3.7%)	
Clinical Stage	Stage I	477675	324608 (68.0%)	227663 (67.7%)	96945 (68.5%)	**<.001**
	Stage II		131474 (27.5%)	93174 (27.7%)	38300 (27.1%)	
	Stage III		21593 (4.5%)	15310 (4.6%)	6283 (4.4%)	
Lymph Node Involvement	1. No lymph node	477675	334962 (70.1%)	234623 (69.8%)	100339 (70.9%)	**<.001**
	2. 1-3 lymph nodes		98948 (20.7%)	70649 (21.0%)	28299 (20.0%)	
	3. 4+ lymph nodes		32997 (6.9%)	23457 (7.0%)	9540 (6.7%)	
	4. Unknown		10768 (2.3%)	7418 (2.2%)	3350 (2.4%)	
Hormonal Receptor Status	1. Yes	477675	477675 (100.0%)	336147 (100.0%)	141528 (100.0%)	-
Surgical Treatment	1. Lumpectomy or partial mastectomy	477675	308552 (64.6%)	215198 (64.0%)	93354 (66.0%)	**<.001**
	2. Total mastectomy		169123 (35.4%)	120949 (36.0%)	48174 (34.0%)	
Adjuvant Radiation	1. Yes	477675	318749 (66.7%)	223919 (66.6%)	94830 (67.0%)	**0.009**
	2. No		158926 (33.3%)	112228 (33.4%)	46698 (33.0%)	
Chemotherapy	1. Yes	477675	142720 (29.9%)	101751 (30.3%)	40969 (28.9%)	**<.001**
	2. No		334955 (70.1%)	234396 (69.7%)	100559 (71.1%)	
Neoadjuvant Chemotherapy	1. Yes	477675	29020 (6.1%)	20722 (6.2%)	8298 (5.9%)	**<.001**
	2. No		448655 (93.9%)	315425 (93.8%)	133230 (94.1%)	

TableS1A. Characteristics of Patients with HER2-Low and HER2-Zero HR+ Resectable Breast Cancer.

**Variable**	**Level**	**N**	**Overall N=477675**	**Her2 Low N=336147**	**Her2 Zero N=141528**	**P-Value**
Adjuvant Chemotherapy	1. Yes	477675	99518 (20.8%)	70992 (21.1%)	28526 (20.2%)	**<.001**
	2. No		378157 (79.2%)	265155 (78.9%)	113002 (79.8%)	
Hormone Treatment	1. Yes	477675	424719 (88.9%)	299887 (89.2%)	124832 (88.2%)	**<.001**
	2. No		52956 (11.1%)	36260 (10.8%)	16696 (11.8%)	
Comorbidity Score	0	477675	397176 (83.1%)	279313 (83.1%)	117863 (83.3%)	0.085
	1		62711 (13.1%)	44345 (13.2%)	18366 (13.0%)	
	2		12833 (2.7%)	9053 (2.7%)	3780 (2.7%)	
	>=3		4955 (1.0%)	3436 (1.0%)	1519 (1.1%)	
Year of Diagnosis	2010	477675	41012 (8.6%)	29381 (8.7%)	11631 (8.2%)	**<.001**
	2011		47603 (10.0%)	34195 (10.2%)	13408 (9.5%)	
	2012		51886 (10.9%)	38728 (11.5%)	13158 (9.3%)	
	2013		59131 (12.4%)	42018 (12.5%)	17113 (12.1%)	
	2014		62747 (13.1%)	44909 (13.4%)	17838 (12.6%)	
	2015		67505 (14.1%)	47416 (14.1%)	20089 (14.2%)	
	2016		72812 (15.2%)	50041 (14.9%)	22771 (16.1%)	
	2017		74979 (15.7%)	49459 (14.7%)	25520 (18.0%)	
